# Special Commentary: My Perspective on Vision and Vision Rehabilitation

**DOI:** 10.1016/j.xops.2024.100532

**Published:** 2024-04-18

**Authors:** August Colenbrander

**Affiliations:** Rehabilitation Engineering Research Center, Smith-Kettlewell Eye Research Institute, San Francisco, California

**Keywords:** Functional vision, Mental Model, Surround vision, Visual functions, Visually guided behavior

## Abstract

Vision is the most powerful sense guiding our interaction with the environment. Its process starts with the retinal image as input and results in visually guided behaviors as output. This paper summarizes insights I gained over >40 years dealing with clinical ophthalmology, visual science, and vision rehabilitation, disciplines that all involve vision, but from different points of view. The retinal image contains 2-dimensional forms that have no inherent meaning. The brain matches this input to stored concepts, to create a Mental Model that is filled with 3-dimensional objects that are meaningful and linked to other senses. Ultimately this leads to the output of goal-directed visually guided behavior. The processes involved are too complex to be covered by a single practitioner. Optimal vision rehabilitation requires teamwork that includes contributions from various professions. It also requires an understanding, as well as possible, of the cerebral processes involved. The visual sciences study mostly the input-driven process from retinal image to visual percepts. Their studies deal mostly with groups and group averages and only occasionally with individual disease conditions. Clinical ophthalmology deals mostly with individuals, rather than group averages. The motto of the American Academy of Ophthalmology reminds us that the end point of patient care goes beyond “preserving sight.” It also includes “empowering lives” by creating the conditions for goal-directed interaction with the environment through visually directed behavior. Traditionally, the study of vision has mainly involved the conscious part of vision, handled mostly in the ventral stream. However, the subconscious part of vision, handled mostly in the dorsal stream must also be considered. This is further stimulated by the demands of computer vision, image processing, and artificial intelligence. Vision rehabilitation traditionally deals with the input side through better illumination and various magnification devices. This is the domain of low vision aids. Increasingly, however, it must also address the output side, and the involvement of other senses (braille, long cane, and talking books). This requires better understanding of the goal-directed higher visual processes. The supplemental material covers the development of numerical scales to quantify not only visual acuity but also visual abilities, and the use of different tests.

**Financial Disclosure(s):**

The author(s) have no proprietary or commercial interest in any materials discussed in this article.

## Background

Vision is the most important sense for guiding our interaction with the environment.

This discussion is based on a presentation I gave when receiving a Lifelong Achievement Award from the International Society for Low Vision Research and Rehabilitation. In it, I shared some of the insights I have gained over >40 years in vision rehabilitation.

In my career, I have been involved in clinical ophthalmology, in visual science, and in vision rehabilitation. Those domains all involve the eyes, but there are significant differences in how they approach the processes that lead from visual stimuli to visually guided behavior. I want to particularly stress aspects that may be overlooked.

## Overview

**Figure 1:**
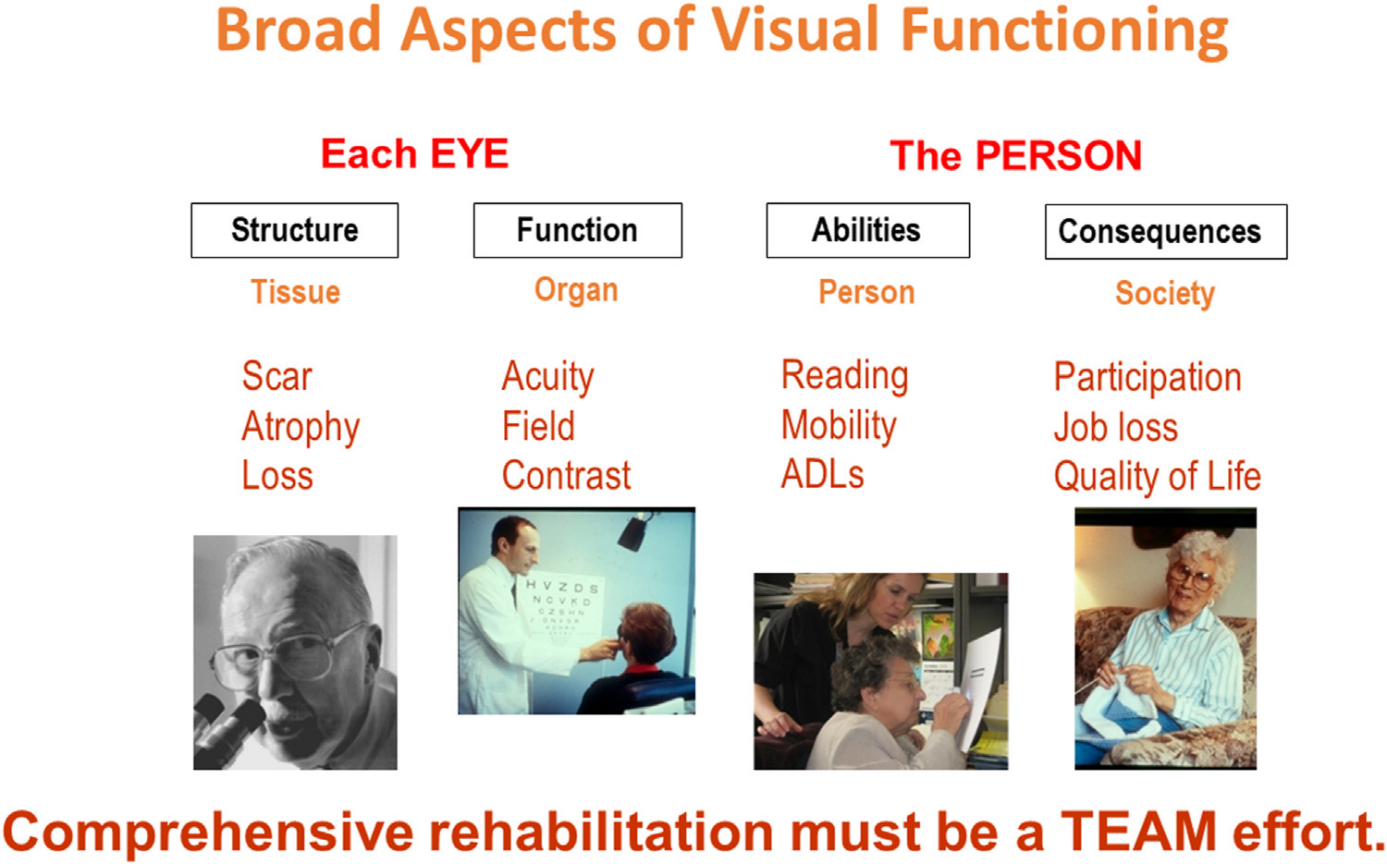
Aspects of visual functioning. ADL = activities of daily living.

For a broad-based overview of our topic, I have found it useful to consider 4 main aspects of vision and visual functioning. The first aspects to be considered are structure and function of the eye. Next are the abilities of the person and the practical consequences of vision loss (Figure 1).

The first aspect focuses at the tissue level, describing aspects such as scarring, atrophy, or loss. Here we need the pathologist to look at the structure of the organ.

However, these structural changes do not yet tell us how well the eyes as a whole actually function. So, we need to broaden our view to include functional changes at the organ level. Here, the clinician can measure functional aspects such as visual acuity, visual field, and contrast sensitivity.

Yet, knowing how each eye functions, does not yet tell us how the person functions. So, we must widen our perspective further to consider the person’s abilities to perform tasks, such as reading, mobility, and activities of daily living. Here, low vision professionals are needed to work with the patient.

Beyond that, we must look at the person in a societal context. Do these changes impact the interaction with the physical and interpersonal aspects of society, causing job loss, or a reduced quality of life? How can we be sure of a contented patient? Since that is the end goal of all our medical interventions and of vision rehabilitation in particular.

From this broad overview, we can already see that the term functioning can be used at many different levels and that no single person can address all aspects comprehensively. Rehabilitation must always be a team effort and the patient must be part of the team. Since I have always promoted this, it is rewarding to note that over my lifetime the role of various rehabilitation professionals has grown significantly and is now considered an essential part of successful vision rehabilitation.

More detailed views of these aspects have been presented earlier.^1^, ^2^, ^3^, ^4^, ^5^, ^6^, ^7^, ^8^, ^9^, ^10^, ^11^, ^12^

## Two Points of View

**Figure 2:**
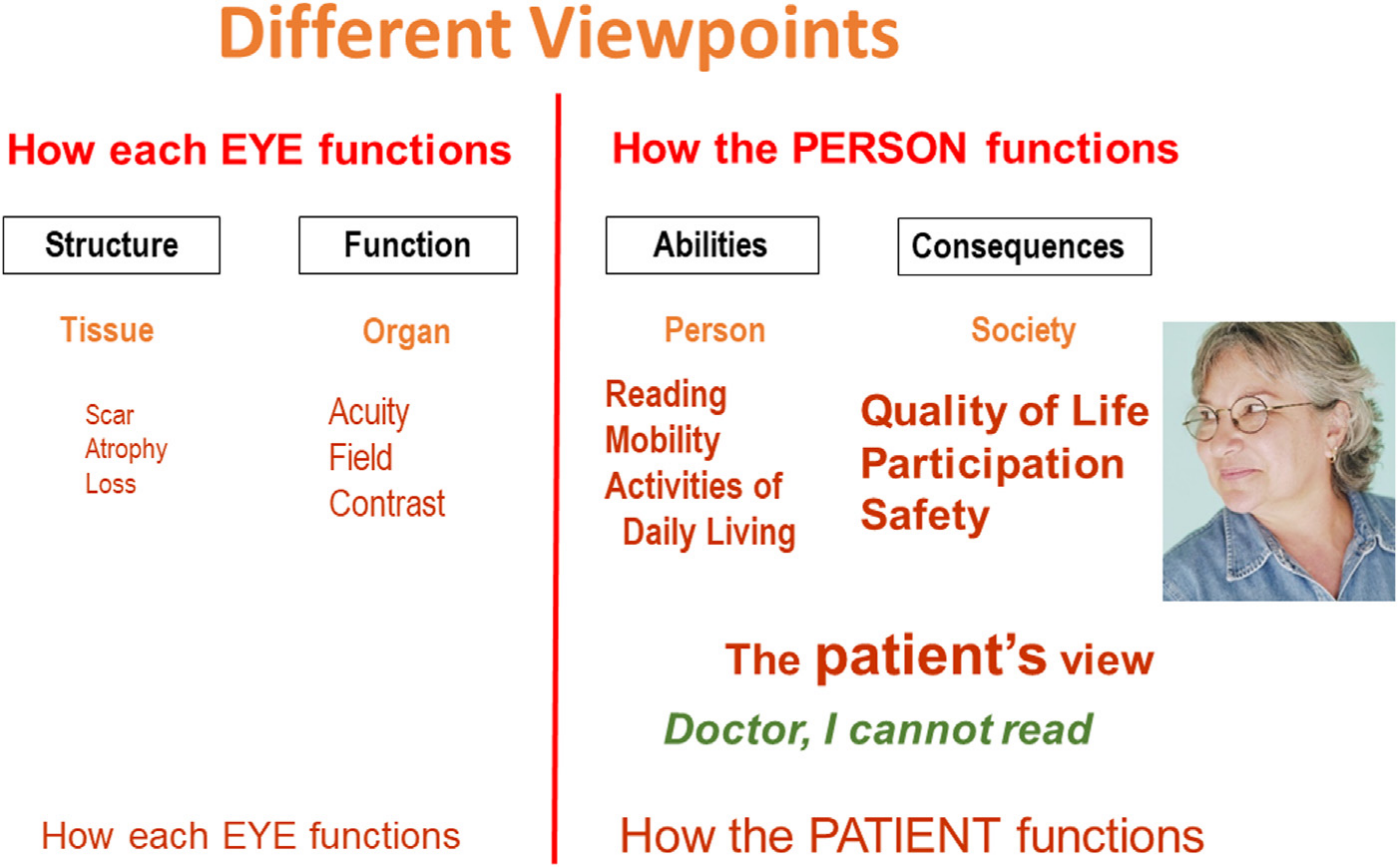
The patients view point.

Comprehensive care needs to consider all aspects; however, the viewpoints of those involved may differ. The point of view of the patient may involve a complaint about an important daily activity: “Doctor, I cannot read.” Her primary concerns are with her quality of life and with her ability to perform daily activities, in short, with the aspects that define how the patient functions in vision-related activities. The aspects on the left side of the diagram are of secondary interest (Figure 2).

**Figure 3:**
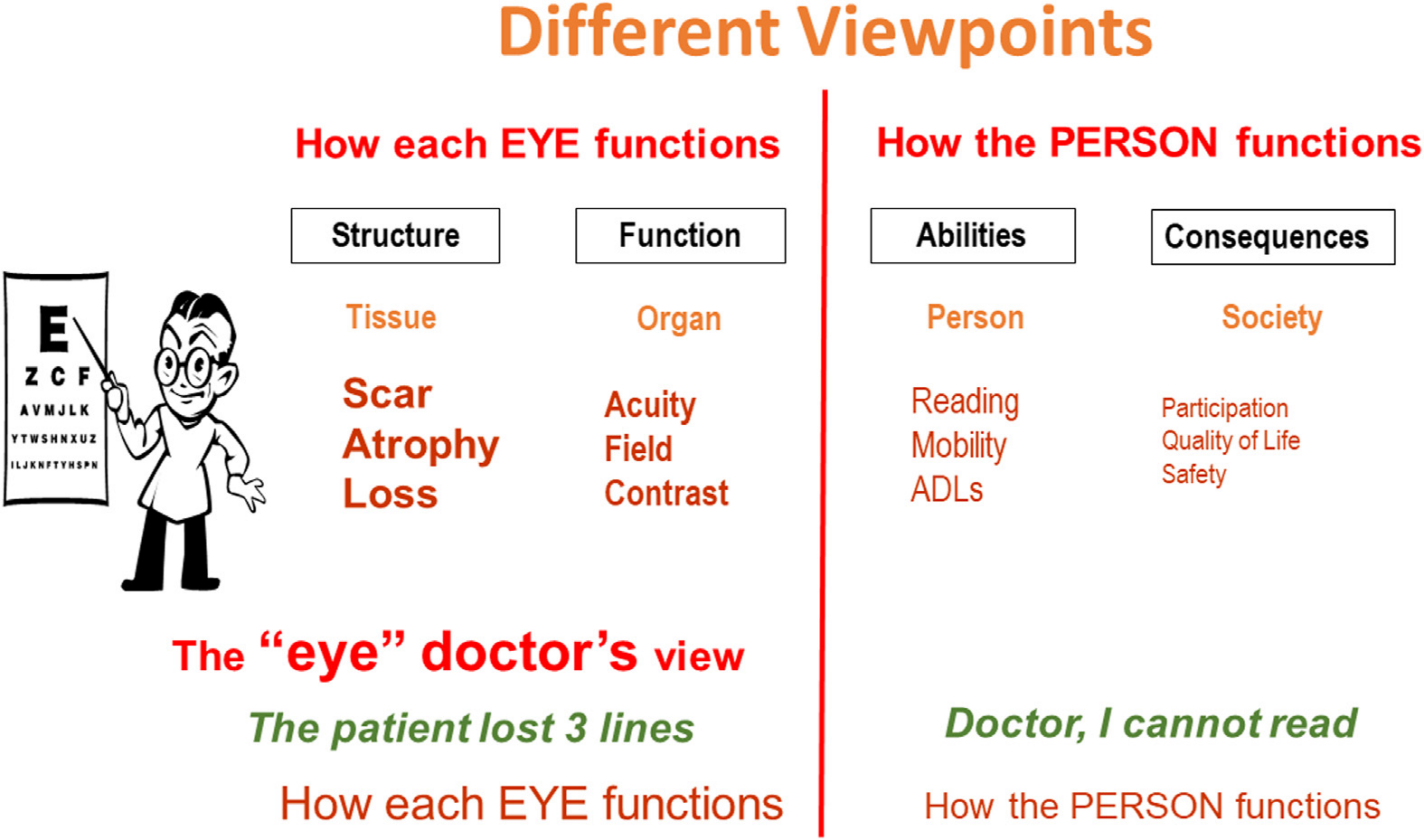
The eye doctor’s view point. ADL = activities of daily living.

Compare this to the eye-doctor’s point of view. He immediately translates the patient’s complaint into a statement about the eyes. “The patient has lost 3 lines.” To him the structure and function of the eye are his primary area of interest, since he is trained to optimize how the eyes function. To him, the aspects on the right side of the diagram are of secondary interest (Figure 3).

This difference between the doctor’s and the patient’s emphasis is the result of them having different objectives. If not recognized, these differences may be a source of miscommunication. On the doctor’s side, the emphasis is on visual functions, such as visual acuity, contrast sensitivity, and visual field, which are important because his objective is to learn more about the underlying causes. For the patient, the primary emphasis is on activities of daily living, since her goal is to cope with the societal consequences.

These different viewpoints are not unique to our profession. Consider the difference between a clock maker and a clock user. To build or repair a clock, the clock maker must know how all components interact. He is less concerned about how the clock will be used. The clock user wants to know when it is time to go to work, to eat, or to sleep. She is less interested in the mechanics.

That both aspects are equally important is reflected in the motto of the American Academy of Ophthalmology. As ophthalmologists, our task is not limited to protecting sight. Equally important is the task of empowering lives. The difference in objectives also helps to explain the subtle differences that may exist in the points of view of clinical ophthalmology and vision rehabilitation.

## Eye Care versus Rehabilitation

**Figure 4:**
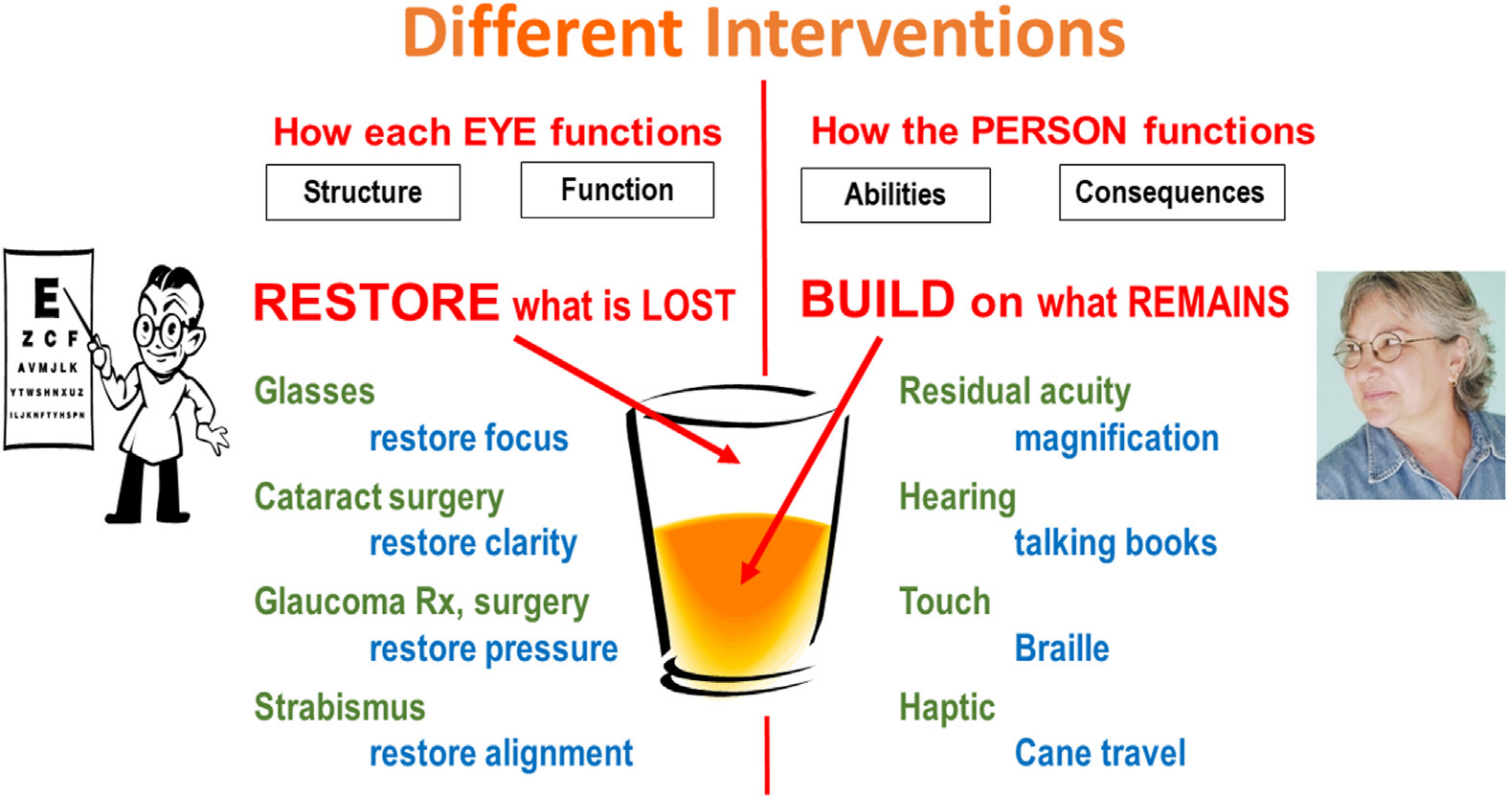
Different interventions.

To expand on these differences, the patient’s condition may be compared to a glass that may be described as half empty, but that can also be considered as being half full. Medical and surgical interventions aim at restoring what is lost. Rehabilitative interventions focus on what remains, because that is the new basis from which performance has to grow (Figure 4).

The required interventions are very different. On the clinical side of the diagram, we see interventions which the ophthalmologist can perform with minimal cooperation of the patient. To classify them, we follow the anatomy of the visual system. For rehabilitation, on the other hand, no success can be achieved without active participation of the patient. Here, we do not use the anatomy, but different sensory modalities as the organizing principle. From these differences in classification, it should be clear that vision rehabilitation deserves its own domain and cannot be considered to be a subcategory under one of the categories for clinical ophthalmology. For rehabilitation, the use of residual visual input is at the top of the list, but the list is not limited to the visual system. Use of other senses as an adjunct to or as a replacement for lost vision must also be considered.

## The Mental Model of the Environment

A familiar opinion is that we see with our eyes and that visual perception results from progressive processing of the retinal image. However, this is not enough, equally important are concepts stored in memory. The retinal image by itself is only a collection of photons that has no inherent meaning, unless matched to concepts, stored in memory. But those concepts in memory have no basis in reality, unless matched to the retinal image. Matching the input from the eyes to concepts stored in memory, results in what I call a Mental Model of the environment.

That Mental Model is built over time and is multisensory; it is the basis for all further processing and eventually for visually guided behavior. Note that when we close our eyes, the retinal image disappears, but the Mental Model persists. Also, the retinal image ends at the ora serrata, but the Mental Model extends around us. The resolution of the retinal input varies markedly with eccentricity, but our perception of the environment features the same resolution throughout. Although we have separate tests for different aspects of the visual experience, our memories always involve multiple modalities. That is why a smell or a sound can evoke a visual memory, or vice-versa.

The memory bank of perceptual concepts that are matched to the retinal image develops and evolves through experience, particularly in the first years of life, but evolves throughout our lifetime.

The matching process determines visual perception, but it is not always perfect:

• In our dreams, the brain can still provide visual imagery without any retinal input.
• If the matching process does not erase all erroneous perceptions, we may experience the Charles Bonnet syndrome.
• When the wrong match is made, we call it a visual illusion.
• Finally, when the proper match is made, it is the resulting Mental Model that leads to the appropriate perception and the appropriate visually guided behavior.

**Figure 5:**
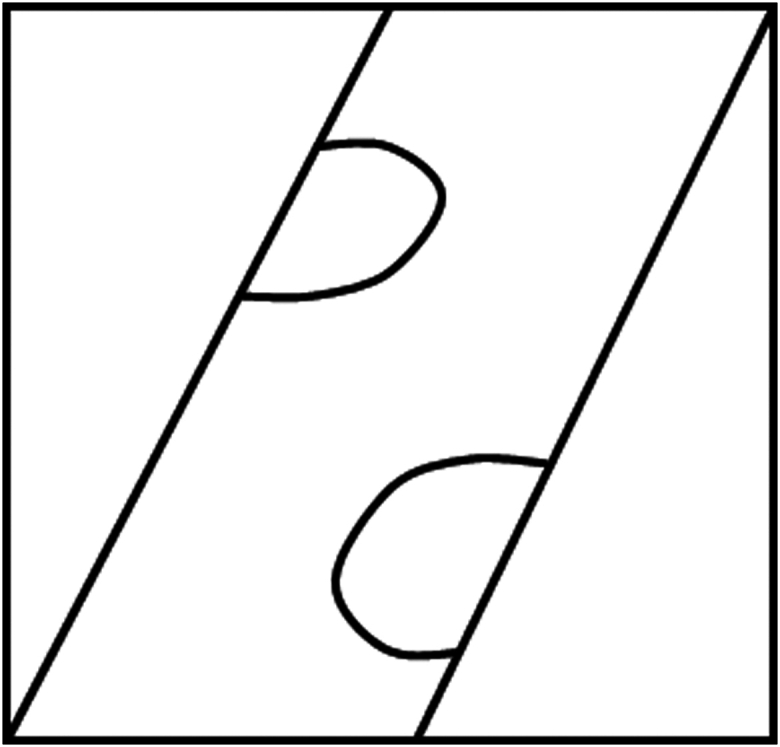
What is this?

When I show you the image in Figure 5, can you understand it?

Probably not. . . . .

**Figure 6:**
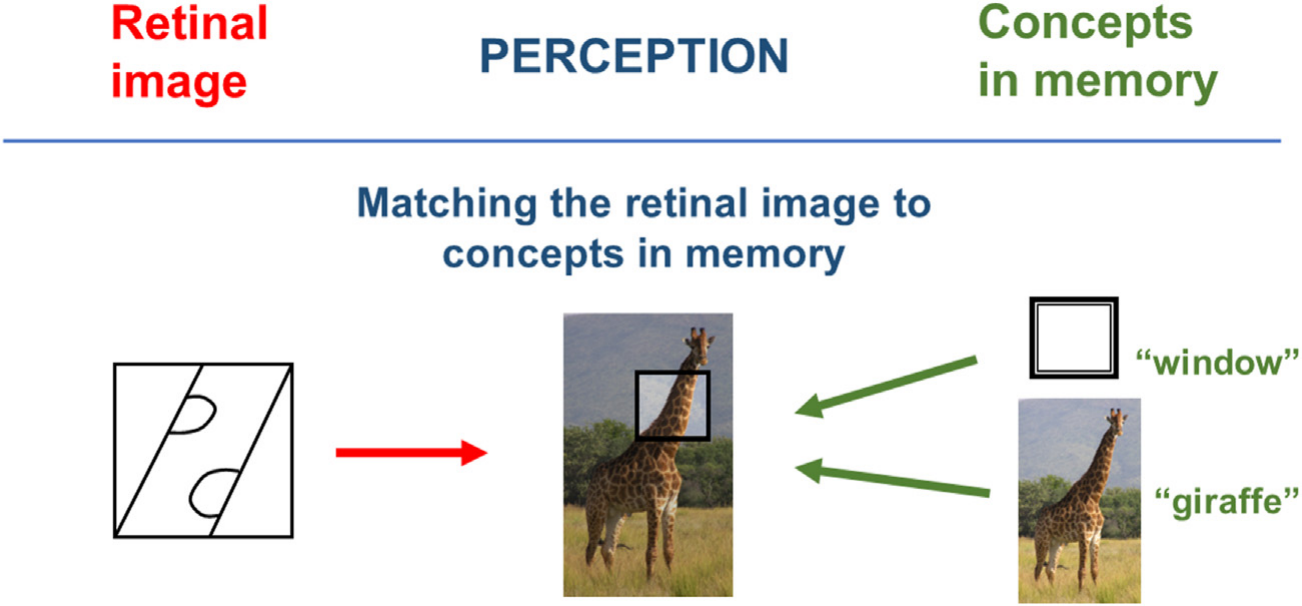
Perception is based on matching two dissimilar sources of input.

But when I tell you that it is a giraffe passing by a small window in the zoo, the image suddenly makes sense (Figure 6).

The auditory input from my verbal comment has triggered the concepts of “giraffe” and “window” in your memory. The visual components of those multisensory concepts can now be matched to the retinal image. Also note that once you have created a Mental Model from these 2 sources, you immediately have access to additional nonvisual information that was not contained in the retinal image but linked to the stored concepts. You immediately know where the head and the body of the animal are; you can even predict how the image will change over time when the giraffe walks on. These examples show that the retinal image alone is insufficient to create a perception. The Mental Model is not exclusively visual. Even the congenitally blind have a Mental Model of their environment.

**Figure 7:**
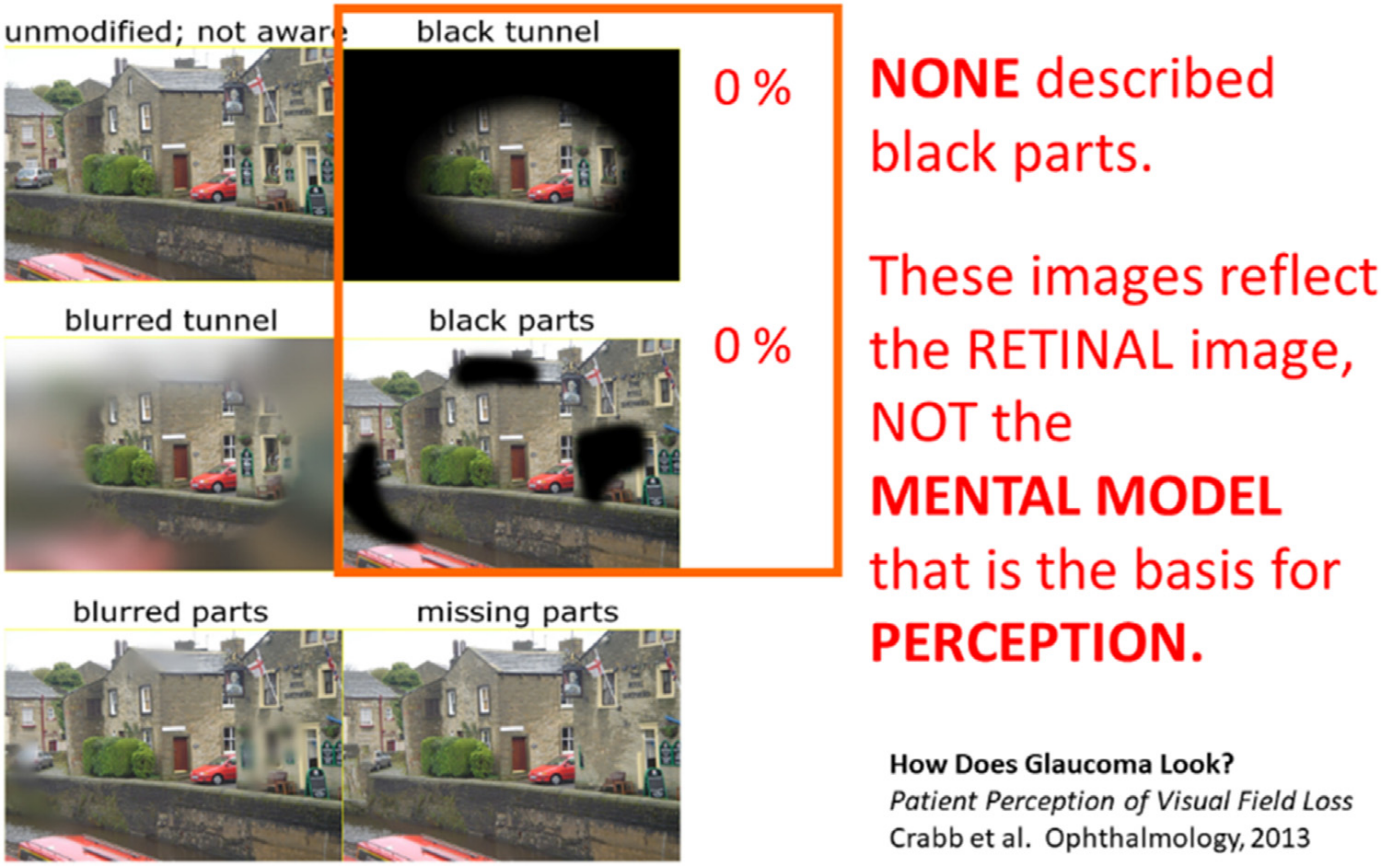
Perception is not based on only the retinal image.

Many other examples can be given. Figure 7 shows the black patches that are commonly shown in textbooks when discussing visual field loss. A British study asked glaucoma patients which of several pictures best resembled their visual experience.^13^ Note that no one chose the images with black patches. This is because the black patches refer to the retinal image, but not to the Mental Model, upon which perception is based.

**Figure 8:**
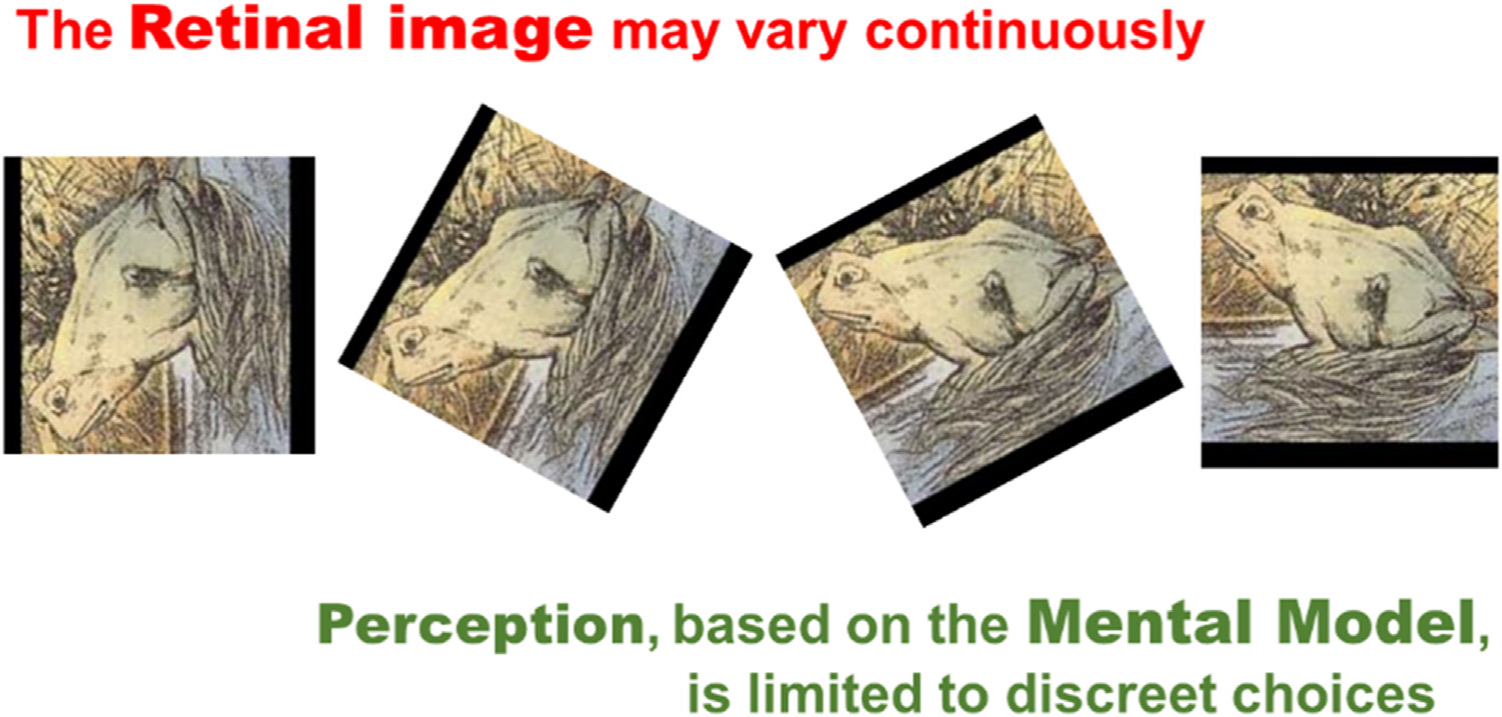
Perception is limited to discrete choices.

Some images can be interpreted in different ways. In Figure 8, the image on the left clearly is a horse. The one on the right clearly represents a frog. But the images in the center show that the orientation is what may determine whether the retinal image is matched to the concept frog or to the concept horse. Note that while the orientation of the retinal image can change gradually, its interpretation only has discrete options. One can alternate between the 2 interpretations, but one cannot perceive an intermediate animal, or 2 animals simultaneously.

These examples demonstrate that the retinal image and the concepts in memory constitute 2 distinct and separate sources. They are very different in nature and cannot be derived from each other. The retinal image consists of 2-dimensional shapes that fill the entire retinal plane like a mosaic. Meaning is added only when the brain interprets these shapes as 3-dimensional (3D) objects in space. Adjacent points in the 2-dimensional retinal image may be interpreted as separate in 3D space. That objects can partly occlude each other in 3D space can give rise to figure-ground separation.

## Reading Ability

**Figure 9:**
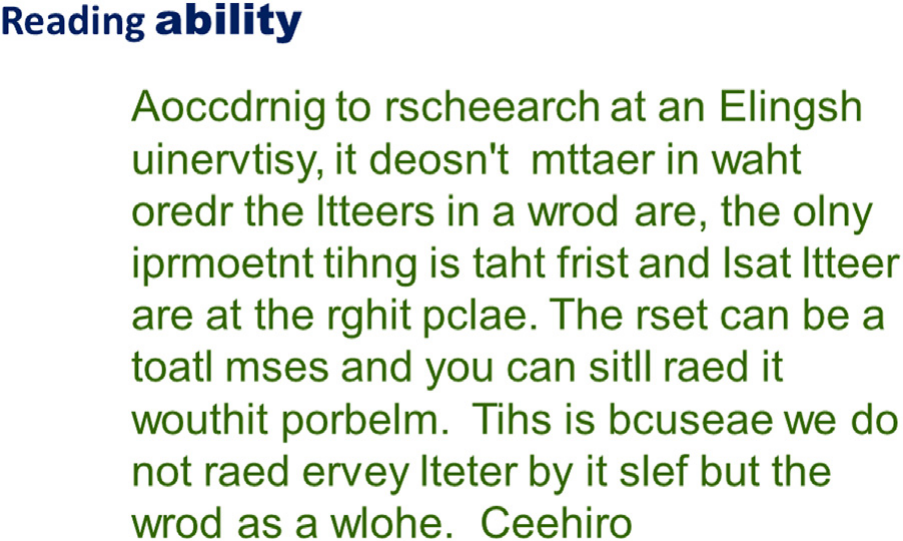
Reading ability involves more than just letter recognition.

This also applies to the reading process, as can be demonstrated by the text in Figure 9, which can be read, even though the letter sequences are severely scrambled. Recognizing a few letters and the context of the story apparently is enough to get to the correct interpretation of the retinal image.

What can be learned from this example? That this text can be read at all proves that fluent reading does not depend on the sequential recognition of all letters. Furthermore, letter recognition is not limited to the center of fixation but can happen simultaneously at multiple retinal locations over a larger area, known as the visual span.

This ability to process information from different retinal locations in parallel provides the visual system with an extraordinary bandwidth, compared with other senses. To differentiate a triangle from a circle by touch, one must follow the contour and count the corners. Vision can recognize the difference in a single glance.

It is often assumed that reading for content starts with recognizing shapes as letters, which are then combined to words, to sentences, and finally to a story. The example shows that this is too simplistic. After lower visual centers recognize several letters simultaneously, higher centers receive what may be compared to an unorganized handful of scrabble tiles. The brain then jumps to generating an educated guess for the word and checks whether this fits the content of the story. The criterion for matching is that the story makes sense, not that all elements of the retinal image are accounted for. This makes sense, since decisions for everyday activities often have to be made, even when the available data are less than optimal.

**Figure 10:**
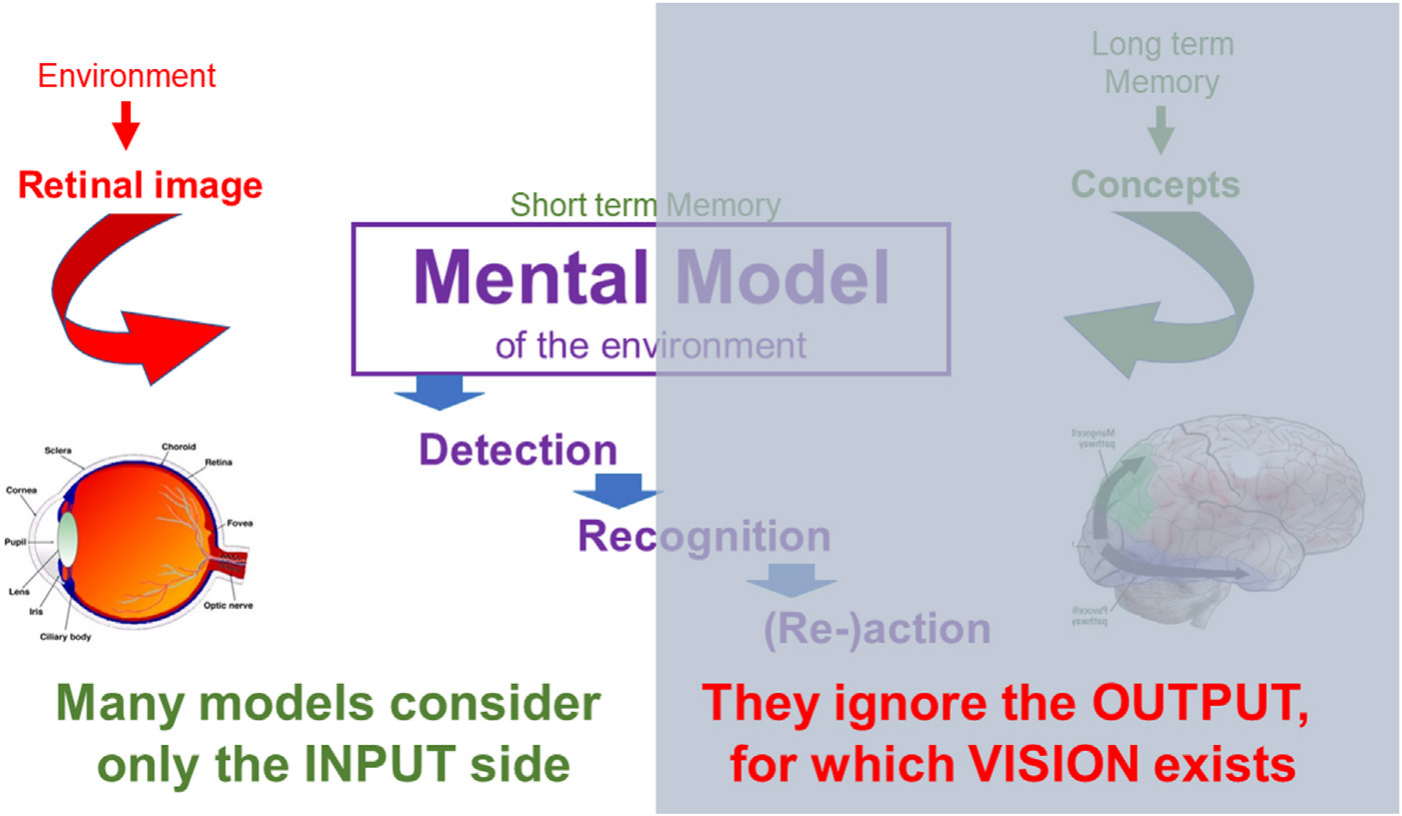
Ignoring the Mental Model ignores the purpose of vision.

Instead of reading for content, we can switch to proof reading mode. For this mode we must retrieve the orthographic spelling of the assumed word from a separate memory bank. This takes time and slows reading down. As low vision practitioners, we know that the reading speed for meaningful text, as on most reading tests, is up to 3× faster than reading of unrelated words. This is another example that shows us that the retinal image provides only 1 part of the information needed for understanding the story (Figure 10).

Discussions that assume that our perceptions are based only on the input from the eyes and on feed-forward processing are incomplete. They ignore that the interpretation of images is the most important function of vision. That interpretation requires feedback and is only possible if the retinal input is matched to a second source of information, that is, to concepts stored in memory. It is this interpretation for which the visual system exists. The output of the visual system is not just a collection of snapshots but a continuing meaningful movie that guides our continuing interaction with our environment.

## Two Modes of Processing

What cerebral processing is needed to move from the Mental Model to visually guided behavior?

**Figure 11:**
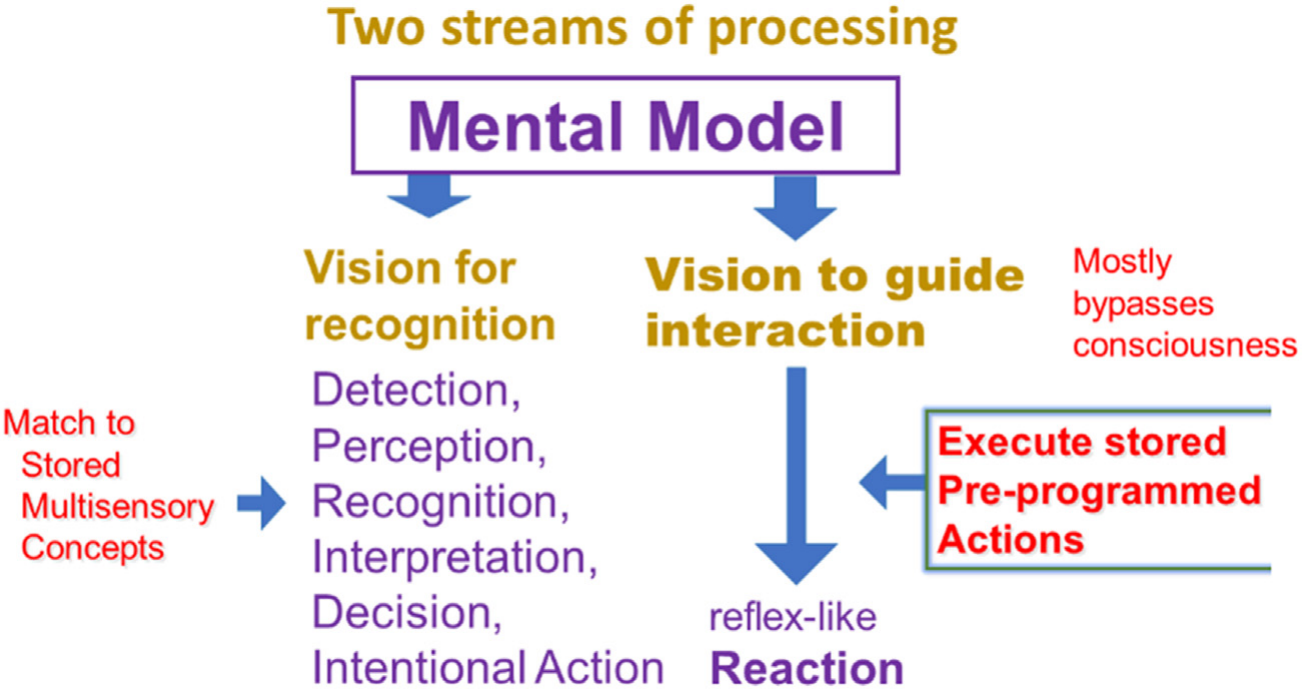
Two streams of visual processing.

So far, we have dealt with the most obvious part of the visual system, the one that leads to object recognition and eventually to intentional action. We have noted that information is drawn from the retinal image, but equally important, from multisensory concepts stored in memory. The process to arrive at intentional behavior involves many steps and decision-making (Figure 11).

However, the visual system provides more than just visual perception. Remember that its final output is visually guided behavior. Behavior involves action. Therefore, we also need to discuss another mode of vision that involves our actual actions. That type of vision is needed, among other things, for navigation and for obstacle avoidance. When we walk through an open door, we do not need to consciously locate each doorpost and then choose to move in the middle. When we read, we do not need to make a conscious decision to move to the next word. The visual system has a subconscious part that does those things for us.

That subconscious or autonomous part of vision can act fast, because it is reflex-like. Like the conscious part of vision, the subconscious part relies on stored information; in this case on stored preprogrammed action patterns.

The simplest demonstration of the 2 different ways in which visual information is processed, is looking through a prism that shifts the retinal image. This has no effect on our conscious ability to recognize objects, but our subconsciously processed ability to accurately reach for objects is severely affected.

Since the 2 modes of processing differ in how they process the same retinal input, as well as in their use of supplemental information and in the type of output, it should be no surprise that the brain has allocated different anatomical areas to the different modes. Anatomically these are known as the ventral and the dorsal stream. However, I prefer to distinguish them by their functional characteristics, that is, as the conscious and the subconscious part of vision.

Recognizing these 2 modes, as well as their interaction, is essential for our understanding of how vision works. Consider picking up an object. The required hand-eye coordination is experienced as a single function. Yet, it needs both systems. The process starts with a conscious decision. Then, reaching for the object, by moving the arm and positioning the hand, is mostly an automated, subconscious process, involving the dorsal stream. It requires absolute spatial coordinates. Opening the fingers just wide enough to grasp the object is also a subconscious process, but it requires only relative coordinates between the hand and the object, more closely related to the ventral stream and to our understanding of the nature of the object. Is it hard or soft, light-weight or heavy?

The interconnections between these 2 streams, and the pathways supporting them, are rarely mentioned in discussions that consider the dorsal and ventral stream as separate entities.

**Figure 12:**
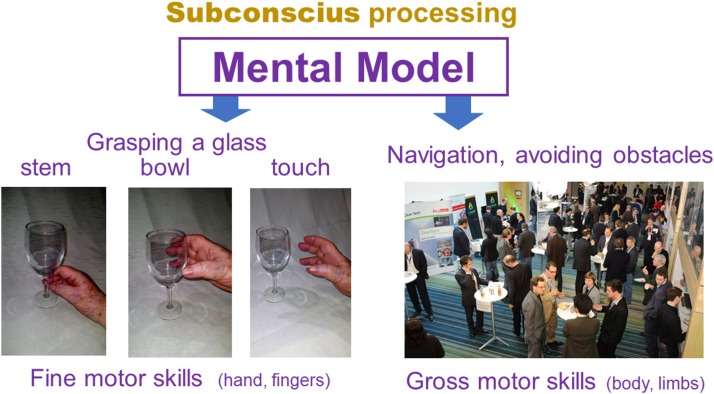
Examples of sub-conscious processing.

Two other examples can further demonstrate the role of these subconscious decisions (Figure 12).

Consider grasping a wine glass. When we want to grasp the stem, our hand opens a little. When we want to grasp the bowl, our hand opens wider. When we close our eyes and rely on touch only, our hand opens even wider. Those changes in hand position depend on visual input; however, they occur completely outside your conscious awareness.

Also, consider moving around at a cocktail party. We give no thought to the fact that we do not bump into people or furniture. Yet, avoiding those collisions requires that the peripheral part of our vision constantly monitors our surroundings and directs our legs and feet to avoid collisions. Those decisions bypass our conscious awareness. This subconscious processing saves our limited attentional resources. Note that the first example relates to fine motor skills, to movements of hands and fingers. The second example relates to gross motor skills, involving the body and limbs.

The subconscious part of visual processing is analogous to how the autonomous part of our nervous system regulates most bodily functions, such as breathing, heartbeat, digestion, etc. without involving our conscious awareness. Conscious and subconscious processing can happen simultaneously; while we are consciously looking for a friend at a party, the subconscious part of our vision prevents us from bumping into obstacles.

## More Complex Rules

The decision rules can be simple or more complex.

As an example, for recognition vision: when a happy face is considered as a single object, its location, size, and orientation do not affect recognition; but a change in the location of details within the object can disrupt the recognition. This means that for object recognition, the information contained in the retinal image is sufficient.

**Figure 13:**
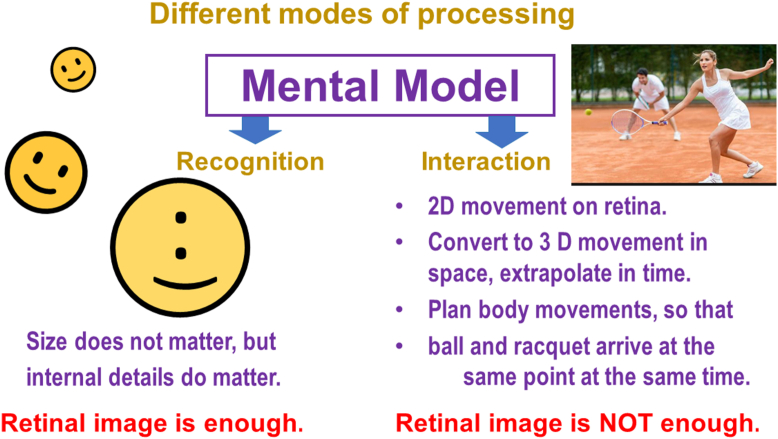
Differences between the conscious and subconscious mode. 2D = 2-dimensional; 3D = 3-dimensional.

For a more complex example of vision for interaction: consider a tennis player (Figure 13).

On the input side, interaction with the ball starts with the movement of the ball’s image on the retina. The brain must then translate that 2-dimensional movement on the retina into a 3D movement in space, and extrapolate that movement in time. Next, the brain must plan complex body movements, and extrapolate these also, so that the ball and the racquet arrive at the same point in space, at exactly the same time, and with the appropriate angle to send the ball in the intended direction. The complexity of this process is such that it could not happen fast enough if it depended on conscious decisions for each of these steps. Conscious processing is not suspended, but it is far too slow to be the controlling force for this kind of visually guided behavior. In the context of vision, the difference between a professional player and an amateur probably depends more on the efficiency of these learned subconscious cerebral processes than on characteristics of their eyes.

We have stated that the ultimate role of vision is to facilitate our interaction with the environment. This clearly is a motor function. Yet, in most discussions of vision and visual perception (beyond the control of eye movements), the unspoken assumption is that the role of the visual system is complete when a perception is formed, which is then handed over to a separate motor system that acts on it independently. Those models are incomplete, because they ignore the need for continuous feedback for our visual-motor interactions.

For recognition vision, the absolute location is often irrelevant. For fine motor tasks, such as writing or drawing, the information about relative position, provided by the retinal image, is sufficient. Vision for interaction, on the other hand, must consider not only information about the position of the image on the retina, but also about the position of the eyes relative to the head, of the head relative to the body, and of the body relative to the environment. The system must then provide output, not only to the hand muscles, but to a variety of other motor systems.

Considering the importance and complexity of these subconscious functions, we may state that conscious vision for recognition is only the tip of the iceberg of visual functioning.

## Differences Between the 2 Modes

To summarize our discussion up to this point, the 2 main modes of visual processing differ in important ways:

• Object recognition requires detail vision. Interactions require spatial awareness.
• For recognition, only limited information about relative location of details is needed. For motor interactions, exact knowledge of location and movement in space are essential.
• Conscious awareness allows conscious decision making. Subconscious reactions are reflex-like. They free up attentional resources by bypassing consciousness.
• Our usual tests of recognition vision deal mostly with stationary conditions, or at least with conditions that require only limited interaction with motor systems.
• Motor interaction involves dynamic stimuli, with motion as part of the input, and with a continuing dynamic visual-motor interaction as output.

The distinction between the 2 modes is not new. It has been referenced under different names. The terms dorsal and ventral stream denote anatomical characteristics. The terms visual functions and functional vision^11^ emphasize that both modes are essential components of the complex phenomenon that is vision. In the context of vision rehabilitation, I prefer to distinguish them based on how they affect visually guided behavior, that is, as how each eye functions versus how the person functions. In orientation and mobility, orientation refers mainly to the recognition aspect, which is input driven, while mobility refers to motor and behavior tasks, which are goal-directed.

## Stimulus-Driven versus Goal-Directed

**Figure 14:**
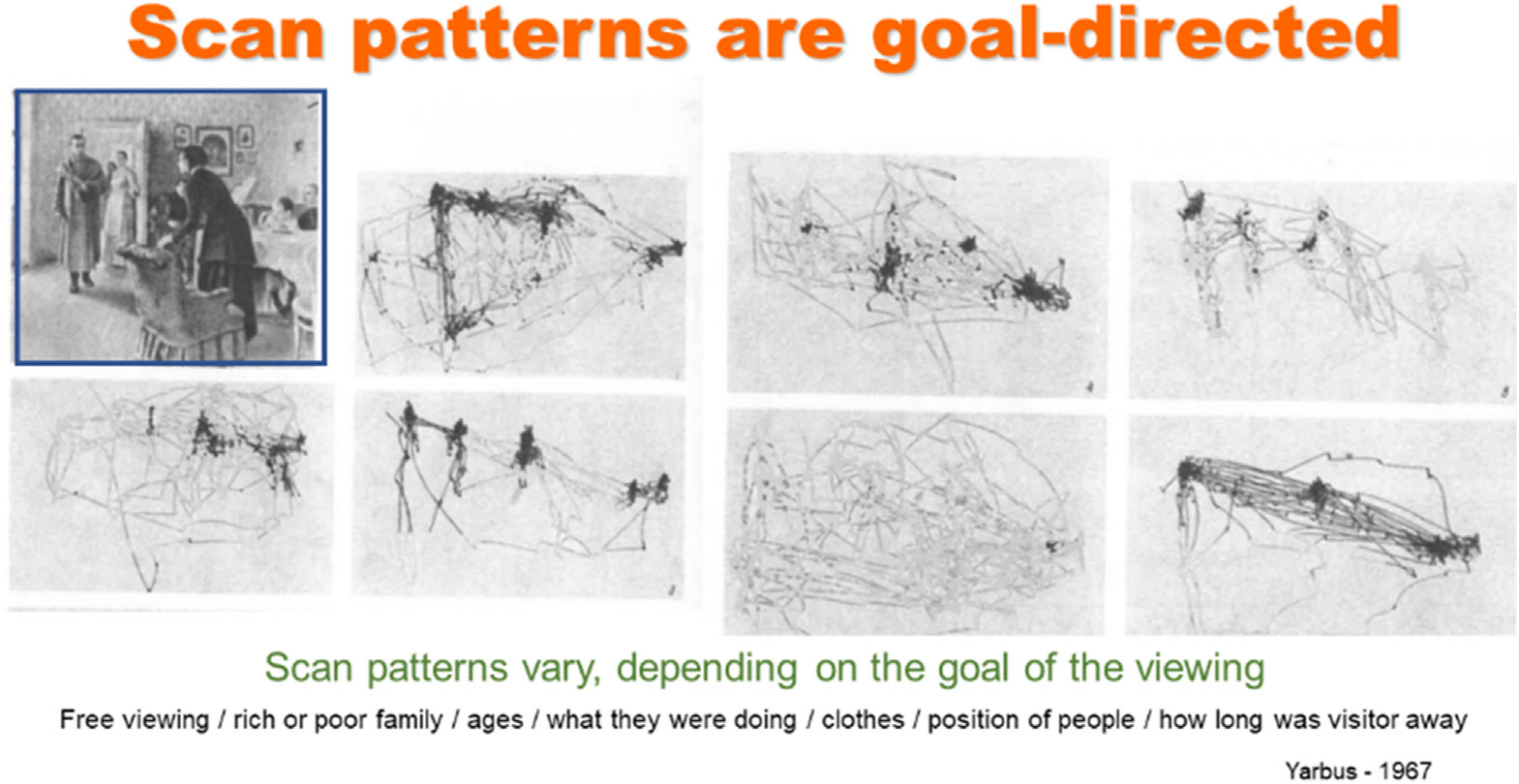
Scan patterns are goal-directed.

The difference between stimulus-driven perceptions and goal-directed actions applies to the recognition stream as well as to the interaction stream. Stimulus-driven connections are often described as bottom-up and goal-directed actions as top-down. We often discuss how salient peripheral stimuli may cause a reactive saccade so that the recognition system can observe the stimulus more closely. We are aware of them because they alert the conscious system. However, those saccades happen only occasionally. We tend to ignore that the vast majority of eye movements are not stimulus-driven but serve the top-down goal of exploring our environment, driven by our own curiosity about that environment (Figure 14).

The images in Figure 14 are half a century old. They display scan patterns recorded from an individual exploring the picture on the left. The stimulus was the same in all instances, but the fact that questions on the mind of the observer differed, resulted in significantly different scan patterns. Most of our testing is stimulus-driven, since that is the aspect that is easiest to control. It is important, however, to remember that the observed responses may also be the influenced by top-down factors, which we cannot completely observe or control.

## Assessing Visual Processing

**Figure 15:**
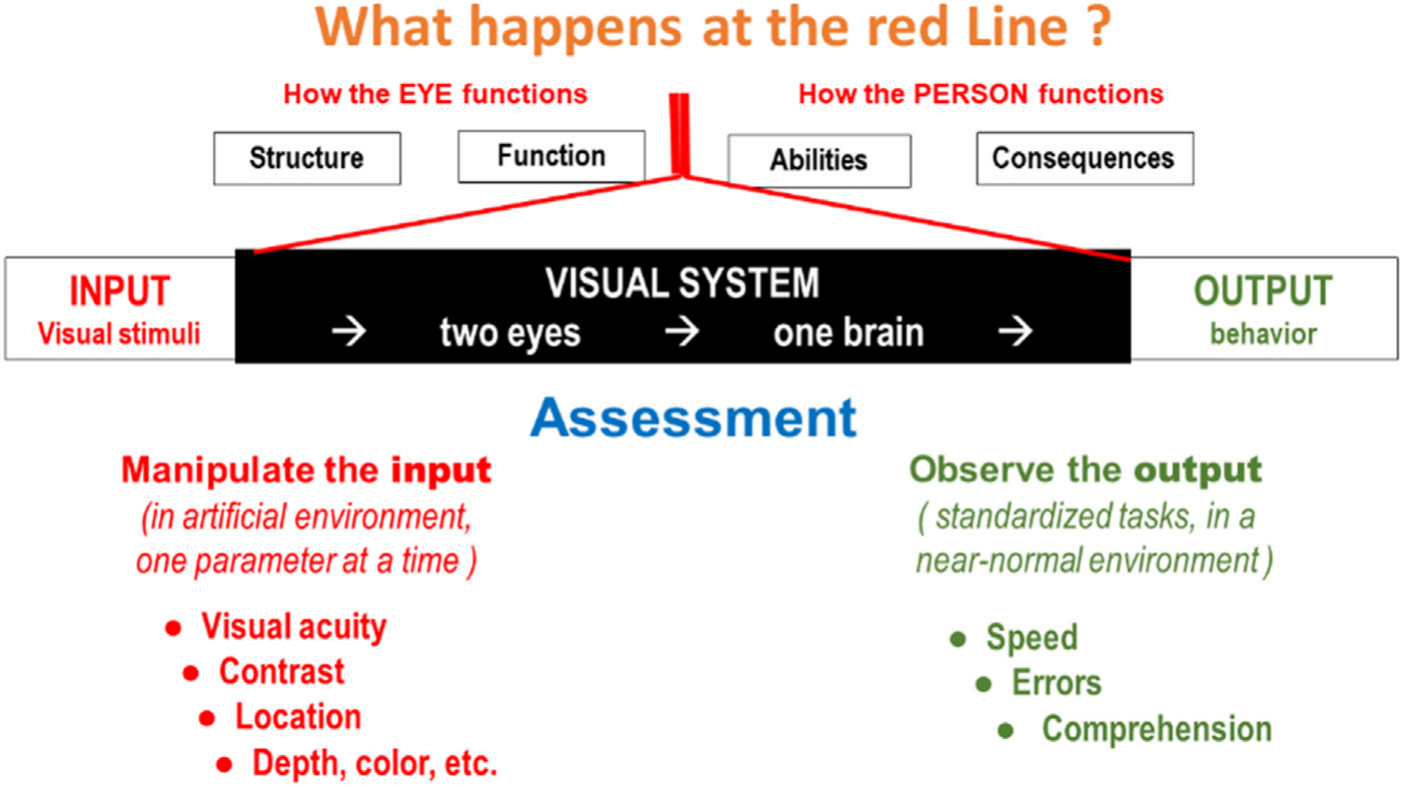
Differences in assessment.

In our initial diagrams there was just a red line in the center of the diagram. Yet, this is where the most important transformations in the visual system occur. When we expand this area to explore how the visual system manages to connect the input of visual stimuli to the output of visually guided behavior, we notice that the input is received through 2 separate eyes, but that a single brain controls the output of visually guided behavior. For a functional assessment, it therefore makes a difference whether we assess the characteristics of vision on the input or on the output side (Figure 15).

**Figure 16:**
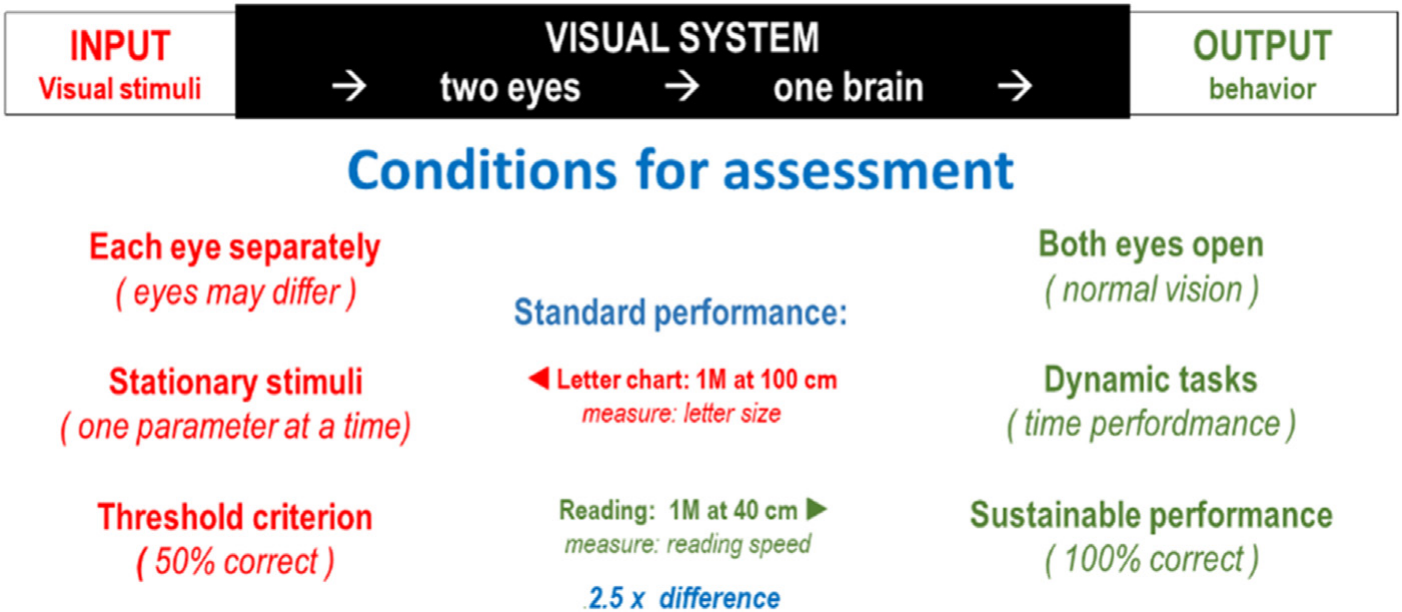
Assessment conditions.

The parameters to be assessed differ significantly (see the supplemental material [available at www.ophthalmologyscience.org] for a more extensive discussion of testing modes):

• On the input side, we can manipulate the input parameters, one at a time. Using an artificial environment, such as a letter chart, allows us to do this one at a time. Thus, we have separate tests for acuity, contrast, location, etc.
• On the output side, we must observe performance of a standardized task, such as reading. Task performance is best characterized by performance speed and performance errors. The scales involved in this assessment are less detailed than is possible when manipulating input parameters (Figure 16).

The testing conditions are different also:

• On the input side, we must measure each eye separately, because the eyes may differ.
• On the output side, we must measure performance with both eyes open, since that is how people live their lives.
• On the input side, we explore threshold conditions, defined as 50% correct. Note that that criterion was chosen because it is optimal for mathematical analysis, not because it is functionally relevant.
• For task performance, we are not satisfied with threshold performance but require sustainable performance, which is near 100% correct. Reading a text and recognizing 50% of the words is not acceptable nor is missing half of the obstacles when walking an obstacle course. The margin between threshold performance and sustainable performance may vary with the task and is often significant. The reference standard for letter chart performance is recognizing 1 M letters (average newsprint) at 100 cm. We measure the letter size. For sustainable reading, we hold 1 M print at 40 cm. and report the reading speed. The difference is a factor of 2.5 × (4 lines on an ETDRS chart).
• On the input side, obtaining detailed responses is generally simple. Since the experience is conscious, we can ask the subjects what they experienced. If we would ask for a conscious response on the output side, which contains many subconscious reactions, we would take the reaction out of the subconscious realm. We therefore have to use indirect methods, such as measuring performance speed and counting the errors. Alternatively, we may use questionnaires that ask about difficulty, quality of life, or other subjective aspects.

Because of these differences, input measurements are preferred in the visual sciences, because they can be more accurate. Output measures are preferred for vision rehabilitation, because they are more relevant to actual daily living tasks.

## Stages for the 2 Processing Modes

**Figure 17:**
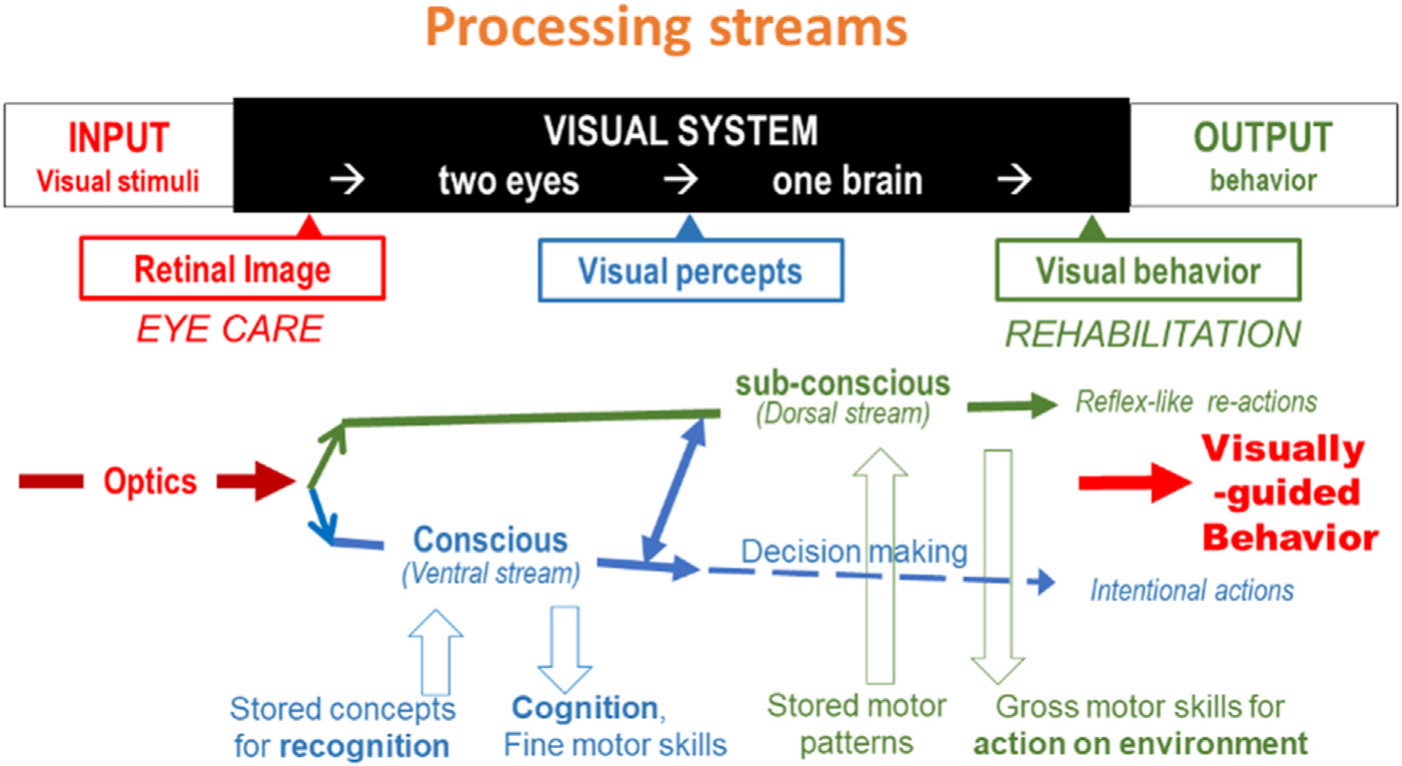
Different processing streams.

In Figure 17 we will combine what we learned about the conscious and the subconscious (autonomous) processing streams and about the different end points and methods for visual assessment.

The 2 processing streams share the same starting point in the retinal image; they also share the same end point in visually guided behavior. In between, however, they follow different routes.

In the conscious, ventral stream, visual processing results in visual perceptions that can give rise to conscious decisions and intentional actions. At the same time, subconscious (autonomous) processing goes on in the dorsal stream, which needs only rudimentary object recognition, but is distinguished by its connection to motor systems that can lead to reflex-like motor actions that bypass consciousness.

Note that these streams can operate simultaneously. As you move around a cocktail party, your ventral stream can consciously look for a friend, while your autonomous dorsal stream prevents you from bumping into people or furniture. The 2 streams are not completely separate. They not only share the same input from the retinal image; they can also exchange information along the way. Discussions that treat the ventral and dorsal stream as separate systems often ignore their interaction.

Both streams draw on stored concepts, but from different sources. For the ventral stream, these are cognitive concepts for recognition. For the dorsal stream, these are stored preprogrammed patterns for motor interaction. The outputs from the 2 streams are also different. The output of the ventral stream is primarily cognitive and is also used for fine motor skills. The output of the dorsal stream is used for gross motor skills and for interaction with the environment.

Let me remind you again that eye care and vision rehabilitation deal with opposite sides of the diagram. Eye care and the visual sciences deal with the left side. Vision rehabilitation deals with the right side and must be knowledgeable about the left side. Vision rehabilitation deserves its own place. It is not just a special instance of eye care.

## Different Domains

Figure 17 can also provide insight into how different groups deal with visual performance.

**Figure 18:**
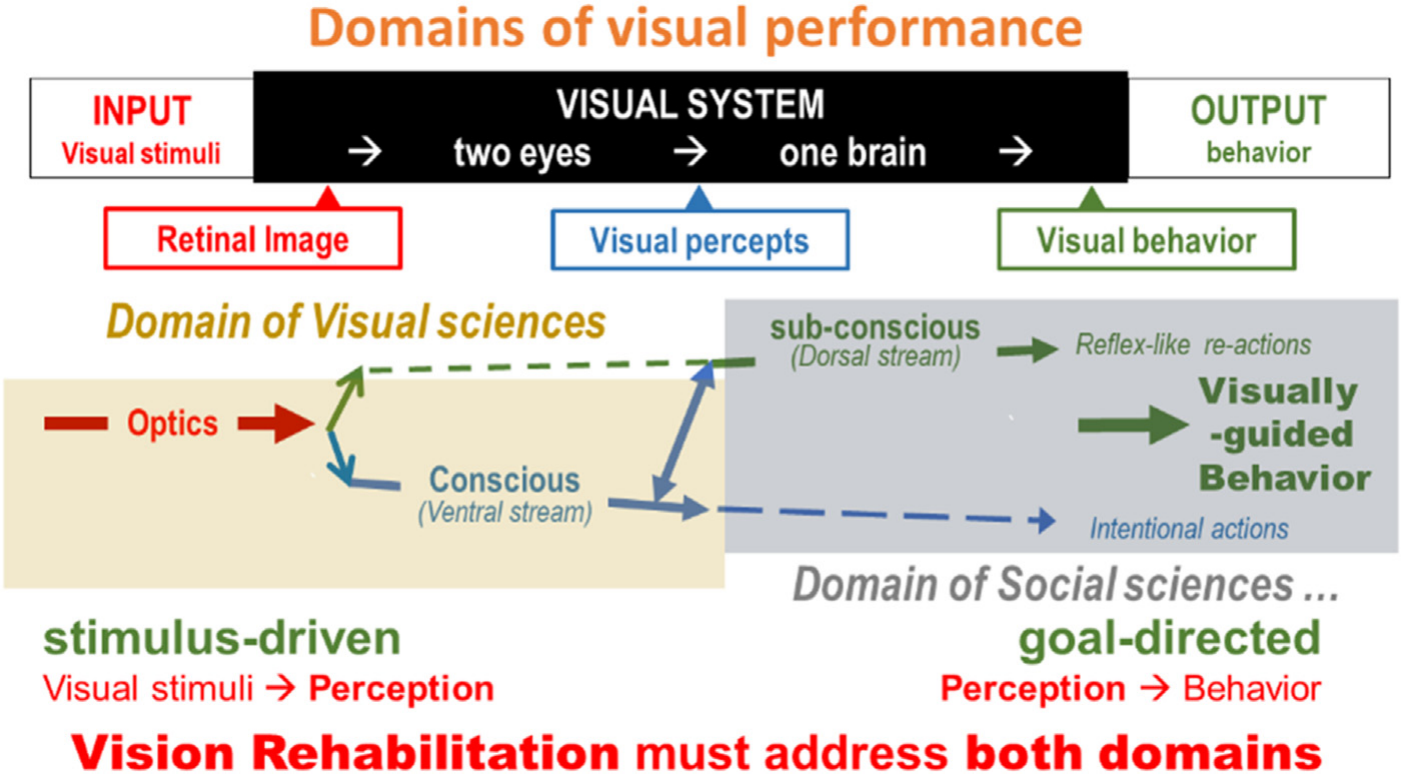
Different domains of study.

Traditional clinical tests and the visual sciences deal mainly with the lightly shaded area on the left (Figure 18). This area is relatively easy to study, since we can control the input of visual stimuli by modifying 1 parameter at a time. The output of this area is also easily accessible, since it involves conscious cognition. We can simply ask subjects about their experience.

For rehabilitation, varying the size of the input stimuli on a letter chart or on a reading test is useful when exploring magnification or illumination options. This is the traditional field of low vision aids. This part of the process is stimulus-driven and leads from visual stimuli to perception. It is the domain of the visual sciences.

Assessment of the gray shaded area on the right is more complex (Figure 18). This part takes us from perception to visually guided behavior. Here, we must deal with the goal-directed part of vision. It is harder to measure, since assessing behavior (the output) is less precise than varying the input. Here, we enter the domain of the social sciences.

This is why the field of vision rehabilitation has evolved beyond the measurement of letter chart acuity and is more extensive than just the provision of low vision aids. This is why we need the involvement of vision rehabilitation professionals, who are trained in areas that medical professionals are not. When dealing with activities of daily living, we cannot stop at the assessment of input parameters. We cannot assume that all people have the same goals, so we need to assess people’s needs through instruments such as an activities inventory^12^ that explicitly asks about the need for and difficulty of various tasks to prioritize them for a rehabilitation plan.

To assess the patient’s behavior, we must resort to indirect methods, such as observed and timed behavior and to questionnaires with graded questions that assess the difficulty of and the need for various tasks and probe the subject’s perceived quality of life. Scientists often characterize such data as “soft.” It is no surprise that visual scientists are reluctant to wade into this domain. They rather leave this to various social scientists. Unlike the visual sciences, vision rehabilitation must address both domains.

The 2 domains also ask for different ways of statistical analysis. The visual sciences mainly deal with groups and with the average response of the average individual. Commonly used statistical methods, such as average, standard deviation, and correlation, use a quantitative approach to deal with these group characteristics and tend to ignore or even eliminate individual outliers. Vision rehabilitation, on the other hand, deals with individuals. Individual rehabilitation plans must reflect how each individual is unique and different from the group average. This approach must emphasize outliers rather than averages and adapt a more qualitative approach. It is useful to remember that, before the advent of modern imaging methods, significant insights into cortical topography were gained from detailed analysis of individuals with unique lesions, not from the analysis of groups.

## Different Stages

**Figure 19:**
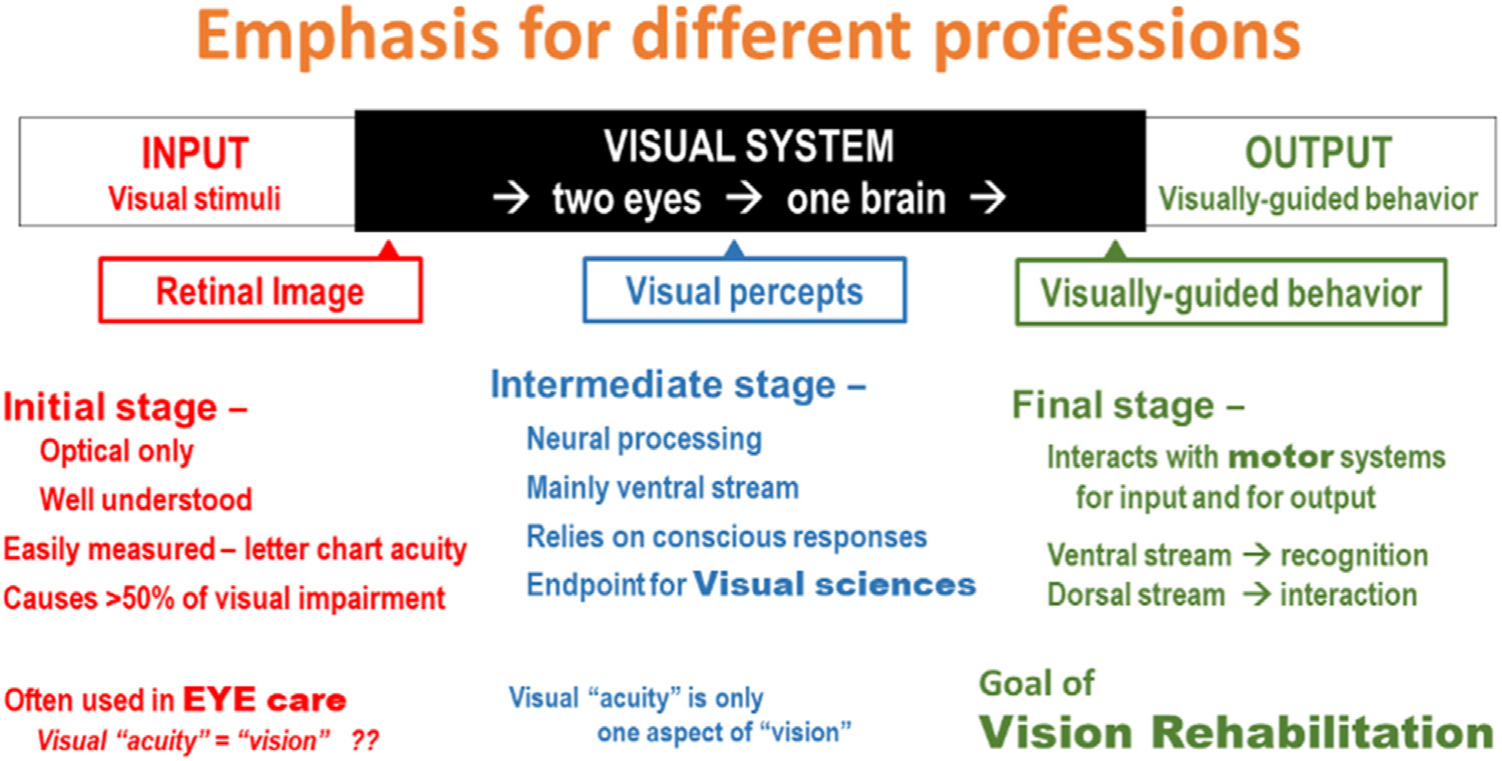
Emphasis for different professions.

Since the transformation from input to output does not occur in a single step, it can be assessed at different points. These points also help to clarify the different viewpoints of the different professions: eye care, visual sciences, and vision rehabilitation (Figure 19).

## Initial Stage—Optical Processing

The first stage of visual processing is an optical one; it produces the retinal image. The optical factors are well understood, and performance is easily measured as letter chart acuity. For clinical use and individual health, letter chart acuity is a good screening test, since it can detect more abnormalities than any other single test. However, letter chart acuity is not diagnostic; other assessments are still needed to determine the cause of the loss. For public health and health statistics, letter chart acuity is important, because it can detect the majority of all visual impairments. The prevalence of deficits in a population provides a reasonable estimate of the visual health of that group.

The emphasis on the retinal image is typical for the eye care professions, who focus on eye conditions. These are the applications for which Snellen developed his letter chart. Because of the use of letter chart acuity for so many other practical applications, the terms vision and visual acuity are often used interchangeably. However, this terminology no longer reflects our expanded view of vision. The relation between measuring letter chart acuity and estimating visual ability is discussed in more detail in the supplemental material (available at www.ophthalmologyscience.org).

## Intermediate Stage—Neural Processing

Since Snellen’s time, the study of vision has expanded significantly. Studying the optical factors remains important, but equally important is the study of neural processing, which starts in the inner retina and proceeds via the visual cortex to higher cerebral areas. This results in an intermediate stage that gives rise to conscious visual percepts, as visual inputs are matched to concepts stored in memory. These stored concepts are largely acquired after birth as the infant learns to interact with the environment. They form an essential component of vision that is different from the retinal image. In this context it is clear that performance on letter chart tests represents only 1 part of the visual experience and does not represent vision in general.

Most recently, these studies are further stimulated by the demands of artificial intelligence, computer vision, and so-called neural networks. True vision requires more information than is contained in the retinal or camera image. Computers still have a hard time simulating the process that produces a Mental Model of the environment by matching the retinal image to concepts in memory. Adequately dealing with goals, rather than just with stimuli, is also a problem.

For much of the visual sciences, visual perception is an end point. At this stage in our discussion, it should be clear, however, that letter chart acuity is only 1 of the many aspects of vision. Snapshots still lack motion, which is an essential element needed for visually guided behavior.

## Final Stage—Visually Guided Behavior

For vision in general and for vision rehabilitation in particular, the most relevant end point is visually guided behavior. This stage is no longer strictly visual, since it also involves motor systems. Processing occurs both consciously (in the ventral stream) and subconsciously (in the dorsal stream). This stage is not separate from motor systems, since it involves continuous feedback loops between vision and motor systems, and interaction between conscious and subconscious decision-making. Vision at this level is no longer a series of snapshots but can best be compared to a continuous movie that unfolds over time and conveys a meaningful story line.

## Conclusions

In conclusion, vision is our most powerful but also our most complex sense. It leads from visual stimuli to visual perceptions and from visual perceptions to visually guided behavior.

We must recognize the intricacies of this system, particularly:

• The retinal image alone cannot generate a visual perception. It must be matched to an equally important, but often ignored, second information source: concepts stored in memory. The result is a Mental Model of the environment. That Mental Model is the basis for all subsequent perception and action.
• Conscious vision is only the tip of the iceberg. Much of visual processing happens subconsciously in the autonomous part of our visual system. This part cannot be assessed by manipulating input parameters but must be assessed indirectly by evaluating task performance.
• Different professionals have different points of view and different strengths in dealing with various aspects.

Comprehensive vision rehabilitation must serve all aspects and always requires team work.

A better understanding of these aspects will give us a better grasp on the challenges of vision rehabilitation, and its relation to other vision-related professional domains.
