# Supplementary material for: Special Commentary: My Perspective on Vision and Vision Rehabilitation
Source: Ophthalmol Sci. 2024 Apr 18;4(6):100532. doi: 10.1016/j.xops.2024.100532 (PMC11373033; doi:10.1016/j.xops.2024.100532)
Supplement: Supplementary materials [file mmc1.pdf]

SUPPLEMENTAL MATERIAL TO: My Perspective on Vision and Vision Rehabilitation

Different Scales for Different Aspects

Measuring vision loss

We have seen how the indiscriminate use of the term "visual acuity" as a way to describe *How each EYE functions* as well as to describe *How the PERSON functions* has caused confusion. Snellen is in part to blame, since he used the Latin term visus (vision) for both aspects. In Europe, the use of visus for visual acuity is still common.

The result of letter chart tests is reported on the visual acuity scale, which is letter size based, but when we average these results, we switch to a different scale, since we are more interested in task performance, than in the average size of the letters seen. Using different terms, such as "acuity" and "ability" for the different aspects can alleviate this confusion, and helps to clarify the difference between seemingly similar tests, such as the reading samples of Jaeger and of Snellen.

Historical development

The second half of the 19th century is often referred to as the Golden age of Ophthalmology. In it, ophthalmology rapidly developed to become the first organ-based medical specialty.

In 1851 Helmholtz invented the ophthalmoscope.

In 1856 Jaeger provided sets of reading samples in various languages. First as an appendix to a book about cataract surgery, later as an independent publication [13]. Jaeger was a clinician interested in *How the PATIENT functions.* He chose reading as a relevant daily task. He did not specify a specific viewing distance for his reading tests, since he cared more about reading performance, than about exact measurement of visual acuity. To identify the print sizes, he used the catalogue numbers from the Vienna State Printing House [14]. When others wanted to print similar samples, they had to do so with locally available fonts. The result was that there is little uniformity in the way various cards use so-called "Jaeger numbers".

In 1861 Donders, who was preparing his epoch-making work on Accommodation and Refraction [15], proposed a more accurate method at a meeting in Heidelberg [16]. As a scientist, he was interested in *How each EYE functions,* and in the function of the refractive system *i*n particular. He asked Snellen, his co-director at the Utrecht Eye Infirmary in the Netherlands, to develop a measurement tool. He also asked one of his students to do a doctoral study on the evolution of visual acuity with age [17].

The next year, in 1862, Snellen published his letter chart [18]. The first major buyer was the British army, which used it for screening of recruits. Among Snellen's innovations were the following.

- He chose a test for distance vision, rather than near vision (reading), because at a sufficient distance the effect of accommodation and the effects of head movements are negligible.
- He used letters, rather than text, which not only reduced the size of the test, but also reduced the cognitive element.
- Since book print, at the time, often involved various fancy fonts, he designed special characters on a 5x5 grid solely to be used for acuity measurement. He called them optotypes.
- Rather than reporting the size of the letters a subject could just recognize, he compared the size seen by the subject to that seen by a standard observer. He defined the latter as someone who could just recognize his optotypes when they subtend five minutes of arc.

- He recognized that the recognizability of characters depends on their angular rather than on their linear size, as used by Jaeger. To specify the angular size two numbers are needed: viewing distance and letter size. He devised the "Snellen fraction" (test distance / letter size), which specifically asks for those two numbers. He always insisted on adherence to this rule. The current use of "Snellen equivalents", which only report the value of the Snellen fraction, regardless of the test distance, is a later development.
- He recognized the importance of standardization, so he based all his measurements on physical units, rather than on local usage. In 1862 there were more than 20 different "feet" used in Europe (he chose Parisian feet). As soon as the treaty of the Meter was signed (1875), he converted to the metric system.

Snellen also published reading samples in several languages, but his main emphasis was on measurement of letter recognition. Jaeger's emphasis was on reading performance.

Measuring letter chart acuity soon became the preferred way of assessing the visual system. The ubiquity of letter chart testing led to the situation where most lay people and even many professionals use the term visual acuity as if it describes all that needs to be known about the entire visual experience.

Letter chart acuity only assesses the retinal area where each letter is projected, which, even for a 20/200 letter, is less than 1 degree. In 1862, this was not a problem, since the study of diseases in the retinal periphery was still in its infancy (the ophthalmoscope was merely a decade old), and since central defocus predicts similar defocus in the periphery. Today, this is a problem, since retinal and other non-optical problems occupy a major part of the average ophthalmologist's time.

In 1868 Green, who had spent time with Donders and Snellen when he traveled Europe after his graduation in Boston, proposed a new chart [19], but his proposals fell on deaf ears. He was a century too early. His chart featured the following.

- He used a strict logarithmic succession of letter sizes, based on $\sqrt[10]{10}$. This series fits well with the decimal system, since 10 steps equal exactly 10x. Its values are convenient since they can be easily rounded to simple numbers, with errors of less than 1%, and since 3 steps are rounded to 2x. This sequence is widely used in industrial standards, and even has its own standard [20]. Jaeger's and Snellen's series were partly logarithmic, but for practical, rather than for mathematical reasons.
- He used proportional spacing and an equal number of letters on the smaller lines.
- He used non-serif letters. At the time, the response was that non-serif letters "look unfinished". A century later letters with serifs were rejected, because they "look old fashioned".

In the next century no substantive changes in letter chart design were made. Letter chart measurements were mainly done in individual offices, so users did not feel a need for standardization.

In 1982 the National Eye Institute proposed standardized charts for its multi-center collaborative studies, of which the Early Treatment of Diabetic Retinopathy Study [21] was among the first. Known by this acronym as ETDRS charts, these charts are now considered an international standard. It is interesting to observe that they implemented all of Green's proposals. They combined the logarithmic sequence of Green, with the layout of 5 letters on every row and proportional spacing, as proposed by Bailey and Lovie [22], and combined this with the letter design proposed by Sloan [23].

Today, it is noteworthy to consider that, because of the lack of an easy method to assess extra-foveal vision, letter chart acuity is still chosen as the preferred method to quantify diabetic retinopathy, a disease that affects the entire retina.

Terminology

We previously discussed that letter chart acuity is not a descriptor of vision in general. Yet, the term "visual acuity" is often used on both sides of the line that separates *How each EYE functions* from *How the PERSON functions*. To avoid this confusion, I prefer to speak about *detail vision* to describe the

recognition aspect of vision in general, and about *letter chart acuity* to describe the use of a specific tool used to assess visual input. Other measurement tools can be used, such as grating acuity or Vernier acuity. Those different tools do not necessarily result in the same numeric outcome. In our discussion, we use the term letter chart acuity as a generic label for all symbol-based charts. On the output side, we can similarly use task-specific test descriptors, like *reading ability.*

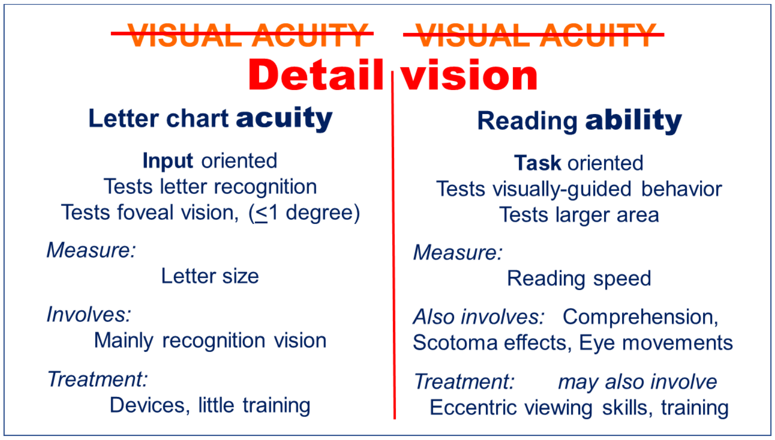

Fig. 20.

Letter chart acuity and reading ability are obviously related. Yet, there are significant differences. Letter chart acuity is a test of detail recognition. The various symbols on the chart have no inherent meaning. Letters are used because they are widely recognized. Reading ability is an example of a more complex visually-guided behavior. Its basic units are words, not letters; what counts most is the meaning of the words in their context (the story), more than the letter sequences.

Considerations for testing

Letter chart testing assesses only vision at the point of fixation. Even a 20/200 letter subtends less than one degree. To test letter chart acuity, we vary letter size, and determine a threshold level for the stimuli. To test reading ability, we judge reading fluency, for supra-threshold stimuli, and can measure reading speed. Reading tests assess a larger retinal area. Letter chart acuity involves primarily visual parameters. Reading also involves cognitive parameters.

When clinicians want to test the optics of the eye and are reasonably sure that there are no retinal abnormalities, testing of letter chart acuity is sufficient. If there are known or suspected retinal factors, as is always the case for vision rehabilitation, using a reading test is more appropriate.

Surround vision

For peripheral vision, a similar distinction between *How each EYE functions* and *How the PERSON functions* can be made. Here, I prefer to use the general term of “surround vision” to indicate the general purpose for which the peripheral retinal image is used.

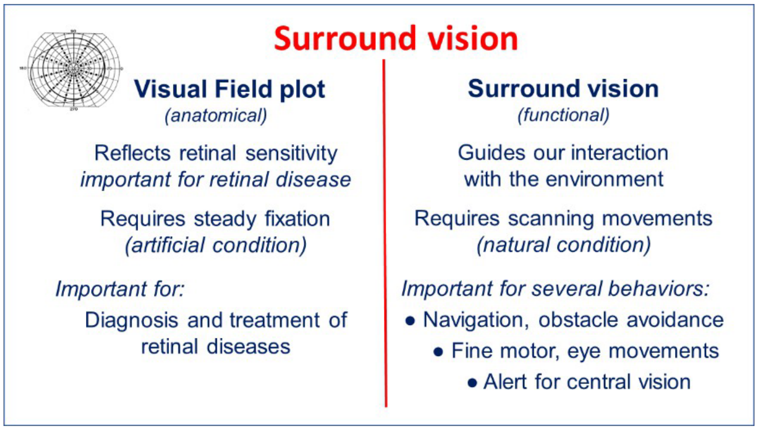

Fig. 21.

The commonly used visual field plot is the outward projection of retinal sensitivities. It is important for the treatment of retinal disease, because it reflects the retinal anatomy.

Most visual field equipment was designed for this purpose. Accurate testing demands the absence or eye movements. This requires the suppression of the natural fixation reflex for salient peripheral stimuli, which is why most patients find the usual visual field tests strenuous.

The term surround vision describes the functional purpose of surveying the visual environment of the person, rather than the anatomical condition of the eye. While testing for visual field plots requires elimination of eye movements, surveying the surrounding space is not possible without scanning eye movements, mostly directed by the subconscious part of the visual system. Tests that allow for these subconscious movements are experienced as far less strenuous.

Surround vision, therefore, is not anatomy based; but rather serves several functional purposes.

- Continuously exploring the environment through conscious or subconscious visual search.
- Control of eye movements, whether for reading, or for exploring the environment.
- Avoidance of obstacles when navigating through the environment.
- Occasionally, triggering a fixation saccade, to alert the central part of vision that a salient peripheral stimulus is worth fixation for more detailed exploration.

Considerations for testing

Unfortunately, there are no simple tests available to explore the functional aspects of surround vision. This leaves a gap between confrontation testing of the visual field, which is simple and quick, but not very accurate, and the use of visual field machines, which are more accurate, but time consuming and expensive, and need a professional operator.

Professional visual field plots are designed for the clinician; they reflect the sensitivity of various retinal areas, but bear little similarity to the patient’s experience, especially, since most of the consequences of visual field loss are only experienced indirectly. Showing the patient successive visual field plots may show them that there are changes over time, but it is not very effective in letting them understand the practical consequences. On the other hand, having the patient look through a simple cardboard tube, gives them a more direct experience. They can still explore their entire environment, but they must consciously make scanning movements with the tube to do so. Thus, they can better understand that a horizontal restriction may cause them to bump into objects; while a lower restriction increases the risk of stumbling. Demonstrations like these may not provide medically useful data, but they encourage patients to take appropriate precautions and may prevent a fall.

Contrast vision

Beyond detail vision and surround vision, the third aspect of vision that may cause problems in daily life is that of contrast vision. Contrast sensitivity loss can aggravate loss of detail vision and loss of surround vision, but its details, whether for diagnostic or for functional purposes, are insufficiently explored. Contrast testing is often included in research studies, but rarely in routine clinical exams, other than for vision rehabilitation. Media opacities may cause contrast loss, as do changes in the receptors (AMD), in neural processing in the inner retina (glaucoma), or changes in the optic nerve. Very different causes may have similar effects, but these have not been used for early detection.

The fact that the eyes can adapt over a wide range of brighter or dimmer illumination, means that the signals that reach the brain contain mainly contrast information and no brightness information. Thus, for the brain contrast is more informative than brightness.

We must, again, make a distinction between measuring threshold differences and measuring functionally significant differences. The usual tests, such as the Pelli-Robson chart, measure threshold contrast across edges. Usually, the letter chart acuity test and the contrast test are presented as separate tests. Comparing the resulting numbers may be meaningful to the clinician, but they mean little to the subject. The Mixed Contrast cards [24] present low and high contrast text side-by-side on one card and allow the subject to make a comparison between the two at supra-threshold levels. This is closer to the effects in real life and more informative to the subject.

One aspect of contrast sensitivity is how gradual changes in brightness are handled. When these changes are due to shadows or to changes in illumination, the visual system tends to ignore them. But under other circumstances, they create the perception of curvature, also known as “shape-from-shading”. There are no clinical tests for the latter phenomenon, and its usefulness in understanding visual processing has not been adequately explored.

Considerations for testing

The high contrast side of a Mixed Contrast chard can be used as any reading test. Comparison to the low contrast side takes very little time and has been found useful for low vision patients. Its usefulness in alerting the patient to possible problems with low contrast objects is obvious, but its use for early detection of various disorders has not been tested yet.

Creating numerical scales

We like to express the result of our observations on numerical scales. Scales for letter chart acuity are the most widely used. If we decide to abandon the habit of using the term visual acuity on both sides of the center line, can we still use the same scale on both sides?

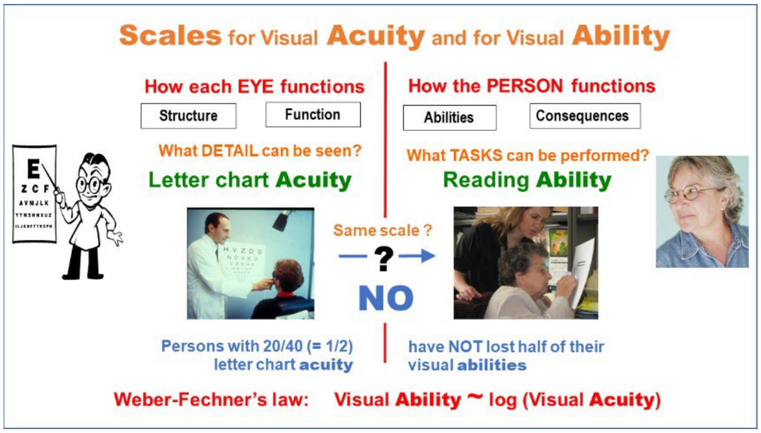

Fig. 22.

The answer is a resounding NO. Persons with 20/40 letter chart acuity have not lost half of their visual abilities. This is where Weber-Fechner's law comes in [25], which states that the *ability* to recognize stimulus differences is generally proportional to the logarithm of the stimulus, in this case to the *acuity*.

Scales for detail vision

There are various scales on which detail vision can be assessed. These scales are mathematically related, but they describe different concepts. To clarify these differences, we will compare them in a diagram, based on the distinction between *How the EYES function,* and *How the PERSON functions*.

In this diagram we place aspects that increase for poorer vision on top, and those that increase for better vision at the bottom.

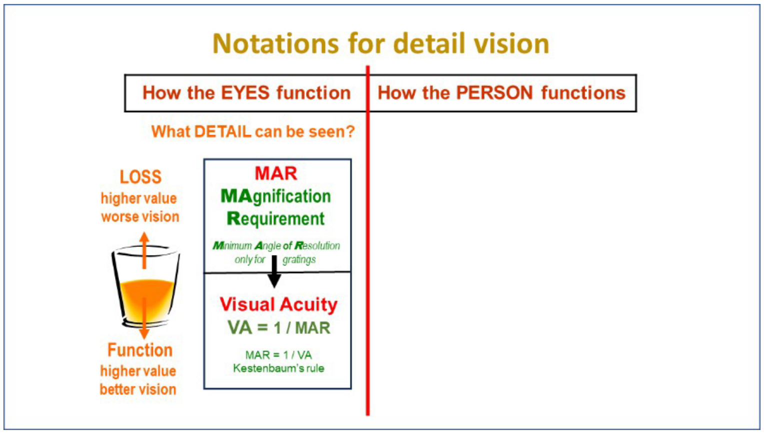

Fig. 23.

To report letter chart acuity for high contrast print, we cancompare the letter size that can just be recognized by the patient to the letter size just recognized by a standard observer.

We call the resulting number MAR, which I interpret as MAgnification Requirement. More MAR means poorer vision; so, we place it on the top of the diagram. For clinical use, however, it is preferable to have a scale where larger numbers indicate better vision. Thus, following Snellen's example, clinicians use the latter option. When doing a letter chart test, they do not report MAR, but its reciprocal and call it visual "acuity" (the sharpness of vision). That scale will be placed at the bottom of the diagram.

VA = 1/ MAR is the simplest way to define letter chart acuity. This definition of VA is simple, but since VA is a reciprocal value, it is not convenient for use in calculations. To prescribe magnification devices, we need to go back to the original MAR value. The expression MAR = 1/ VA, known as Kestenbaum's rule, states that the reciprocal of the letter chart acuity value is a good estimate of the minimal magnification required for reading. Many people use Kestenbaum's rule without realizing that it simply is the reciprocal of the definition of visual acuity.

Some may comment that MAR is commonly explained as "Minimum Angle of Resolution". This, however, is a left-over from the use of gratings to test lenses. For gratings, the width of the black and the white areas is equal, so the stroke width is the only relevant parameter. For letters, the black stroke width can be standardized, but the white areas are highly variable. For letters, the *letter size,* rather than the *stroke width,* determines recognizability. Furthermore, human recognition, as discussed earlier, involves more than just optical imaging. Since resolution only refers to optics, its use in vision might be interpreted as excluding non-optical vision loss, such as amblyopia.

Scales for ability

On the other side of the center line, we must ask about the person's ability to perform *tasks*. The resulting ability scales are needed for a wide variety of purposes. We may need to quantify a person's ability to meet job requirements, or we may need to specify progress in rehabilitation, or we may want to determine disability benefits, such as for workers compensation, or we may need to check compliance with other eligibility requirements, such as for a driver's license.

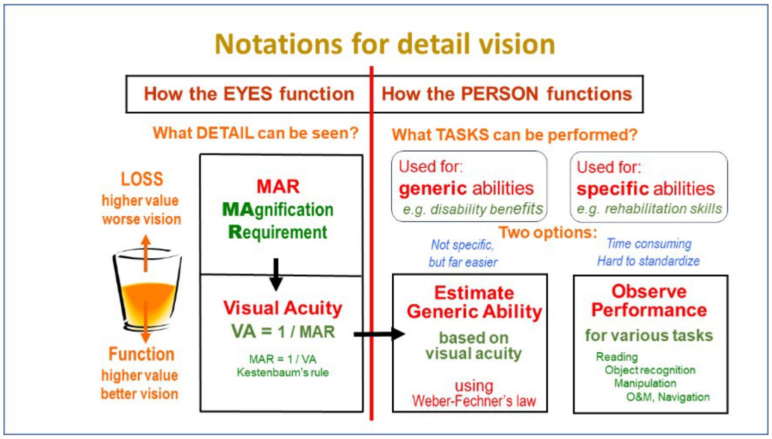

Fig. 24.

At this point, we have two options, we can *observe* actual task performance for various tasks, or we can *estimate* the performance, based on the measured letter chart acuity. Compare: an on-the-road driving test to a test of letter chart acuity in the DMV office.

This distinction is important for understanding various regulatory requirements. For instance, the common requirement of 20/40 for a driver's license is not based on observation. It defines a safety margin, based on the unspoken assumption that someone who can read 20/40 on a well-lit stationary letter chart, can still be a safe driver in a moving vehicle, after dark, in the rain. This may be true for some people, but not for others. For professional drivers the requirements are often stricter, not because they drive in a different environment, but because a wider safety margin is warranted. That this simplification is so widely used, is a side effect of ignoring the distinction between the left side and the right side of the diagram.

The difference between letter chart acuity and task performance is also an important factor in choosing a measurement method. Observing a person's actual abilities is the most informative. It is needed when training people for specific skills, such as for athletics. In vision rehabilitation, it should be used when training people in the use of specific assistive technology, such as cane travel, Braille, or sophisticated electronic devices. However, this method is time consuming, since the number of potential tasks is large, and each observation takes time.

So, for general rules, especially in administrative settings, a performance *estimate* is often used by applying Weber-Fechner's law to the measured letter chart acuity. This method is less accurate, but it is far easier and less time consuming. Use of these estimates for disability benefits that depend on the amount of loss, as in the AMA Guides, is often defended because use of a letter chart is more objective and more generalizable than observer judgements. Note, however, that this method only gives us only a generic estimate for the performance of generic tasks.

Simply applying Weber-Fechner's law to a letter chart acuity value results in a log(VA) value, which is not user friendly, because of its decimal values and its negative range. This problem can be overcome by multiplying the value by 50 (to remove the decimals) and by adding 100 (to bring it to a positive range). The result is known as Visual Acuity Score or VAS, on which 20/20 = 100. Such ability estimates are commonly reported on a percentage scale.

The VAS scale does not require the use of any specific chart, but is especially applicable to ETDRS charts. On ETDRS charts, the VAS scale is a letter count scale, anchored at 20/20 = 100%, which places 20/200 at 50%, and 20/2000 at 0%.

This scale, was recommended by the International Society for Low Vision Research and Rehabilitation (ISLRR, 1999) [26] and by the International Council of Ophthalmology (ICO, 2002) [27]. Since 2001, it is used in the AMA *Guides* [28] to calculate visual ability estimates for workers compensation cases.

Note: the original ETDRS protocol also used a letter count score, but that score was protocol specific. It was anchored at 0% = inability to see the largest letters of the 4m chart at the shortest distance (1 m) used in the protocol. That score placed 20/20 at 85%. The VAS score is independent of any protocol and 20/20 = 100% is more intuitive.

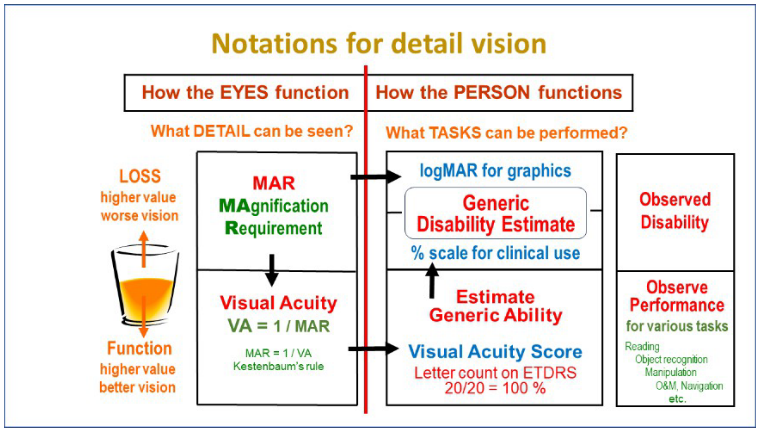

Fig. 25.

This leaves the upper right quadrant, which represents an estimate of visual ability *loss*, also known as visual *dis-ability*. Relating it to the ability rating, we can simply state that disability = 100% – ability. This option is the method used for most legal and administrative purposes, as in the AMA *Guides*.

Alternatively, visual scientists often use the logMAR value, which is directly based on MAR. This mathematically simple formula is widely used to calculate and graphically display disability estimates in their studies. However, its decimal values are not intuitive for clinical use, and relating them to the commonly used letter chart acuity values involves awkward formulas, like $VA = 1/ 10^{logMAR}$.

The use of expressions such as “letter chart acuity as logMAR” demonstrates that the true meaning of these parameters is frequently misunderstood. Letter chart acuity and logMAR are both derived from MAR, but with different formulas for different purposes. VA = 1/MAR describes the sharpness (acuity) of the retinal image; logMAR = log(MAR) estimates the loss or lack of generic visual abilities of the individual. These two uses should not be mixed. Compare this to how speed, time and distance are mathematically related. Yet, this does not justify us to use time units to specify a distance, even though this is often done colloquially.

It is sometimes assumed that the decimal values of the logMAR notation make the reported values more precise or more reliable. This is a mistake. Whichever scale is used, the reported values are no more reliable than the MAR value on which they are ultimately based. Variations in the viewing distance are the most common cause of inaccurate letter chart measurements.

Clinical notations

For clinical use we can simplify the diagram.

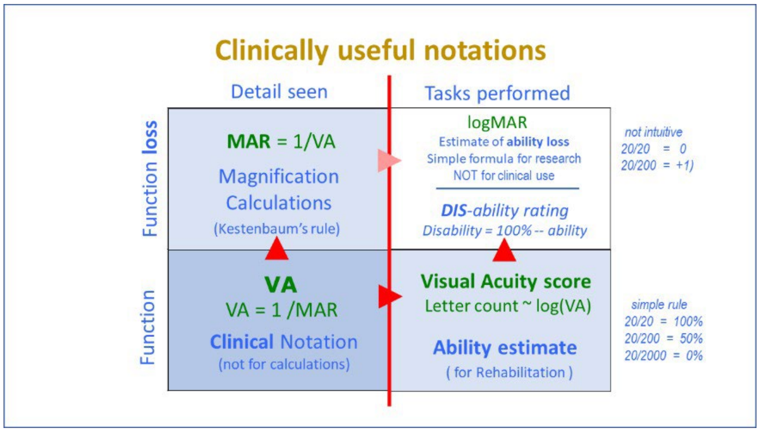

Fig. 26.

MAR (MAgnification Requirement) is the value actually measured with letter charts. Although this value is useful for calculations, it is rarely recorded as such in medical records

For clinical use, letter chart acuity remains the preferred notation. However, since VA is a derived value (VA = 1/MAR), it is not the most useful for calculations.

For calculations about magnification, we have to retrace our steps to what is actually measured, that is to the MAR value. The simple relation between MAR and VA is known as Kestenbaum's rule.

The acuity value also is not fit to serve as an estimate of task performance. To estimate ability, we need to apply Weber-Fechner's law, and use a logarithmic scale. The VAS scale estimates the generic visual abilities of the person. It belongs in the lower right quadrant. It has an easily remembered relation to letter chart values, and on ETDRS charts it is a letter count scale, anchored at 20/20 = 100%.

For the top right quadrant, expressing disability as 100% – ability is the simplest solution. It is used for most administrative applications.

The logMAR scale provides a mathematically simple alternative, based directly on MAR, but for clinical use it is not intuitive, and relating it to VA results in awkward formulas, such as $VA = 1/10^{logMAR}$.

Comparing various scales for detail vision

The following table compares various scales.

Scales for vision-related functioning

How the EYES Function (acuity)				How the PERSON functions (ability)				Ranges
MAR	VA (US)	VA (6m)	VA (dec)	logMAR	Log(VA)	Visual Ability	Visual Dis-ability	
0.8 x	20/16	6/4.8	1.25	–0.1	+0.1	105 %	0 %	Normal range
1.0 x	20/20	6/6	1.0	0	0.0	100 %	0 %	
1.25 x	20/25	6/7.5	0.8	+0.1	–0.1	95 %	5 %	
1.6 x	20/32	6/9.5	0.63	+0.2	–0.2	90 %	10 %	
2.0 x	20/40	6/12	0.5	+0.3	–0.3	85 %	15 %	Mild loss
2.5 x	20/50	6/15	0.4	+0.4	–0.4	80 %	20 %	
3.2 x	20/63	6/18	0.32	+0.5	–0.5	75 %	25 %	
4.0 x	20/80	6/24	0.25	+0.6	–0.6	70 %	30 %	
5.0 x	20/100	6/30	0.2	+0.7	–0.7	65 %	35 %	Moderate loss
6.3 x	20/125	6/38	0.16	+0.8	–0.8	60 %	40 %	
8.0 x	20/160	6/48	0.125	+1.0	–1.0	55 %	45 %	
10 x	20/200	6/60	0.10	+1.1	–1.1	50 %	50 %	
12.5 x	20/250	6/75	0.08	+1.2	–1.2	45 %	55 %	Severe loss
16 x	20/320	6/95	0.063	+1.3	–1.3	40 %	60 %	
20 x	20/400	6/120	0.05	+1.4	–1.4	35 %	65 %	
25 x	20/500	6/150	0.04	+1.5	–1.5	30 %	70 %	
32 x	20/630	6/180	0.032	+1.6	–1.6	25 %	75 %	Profound loss
40 x	20/800	6/240	0.025	+1.7	–1.7	20 %	80 %	
50 x	20/1000	6/300	0.020	+1.8	–1.8	15 %	85 %	
63 x	20/1250	6/380	0.016	+1.9	–1.9	10 %	90 %	
80 x	20/1600	6/480	0.0125	+2.0	–2.0	5 %	95 %	Near-blindness
100 x	20/2000	6/600	0.010	+2.2	–2.2	0 %	100 %	
	- - -	- - -	- - -	- - -	- - -	- - -	- - -	

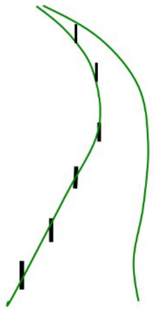

Fig. 27.

The first column lists the Magnification Requirement (MAR) as measured with a letter chart. These values represent a linear (arithmetic) scale.

The next three columns list the clinical notations used in various countries. Note that these are Snellen *equivalents*, not Snellen *fractions*, since they do not indicate the actual viewing distance and the actual letter size. The same notation is used for distance and for near acuity. In the USA, the numerator is standardized to 20, since Snellen originally used a viewing distance of 20 Parisian feet. In Britain the numerator is standardized to 6, since 6 m approximates 20 ft. In Europe the decimal equivalent is often used.

Use of the decimal notation may lead to adoption of a decimal series of letter sizes. Note that this violates Weber-Fechner's law and the logarithmic progression, and leads to awkward ability values.

The next columns compare these values to visual ability estimates. Here we change from a linear (arithmetic) scale to logarithmic (geometric) scales. The logMAR scale provides an estimate of ability loss for graphical displays; but is not intuitive for clinical use. On this scale the most common acuity value for young eyes (20/16) is represented with a negative value. The log(VA) scale (log(VA) = log(1/MAR) = – logMAR) provides ability estimates, but its negative values are not intuitive either. Therefore, the ability scale is usually transformed to a % scale (VAS). The % notation is more intuitive, since it rates 20/20 as 100%, and since the % symbol clearly identifies it as different from acuity values.

The last column indicates that although a % scale has a potential of 100 values, but that level of detail is not realistic for most purposes. For letter chart acuity the 20 values shown are realistic for single measurements; finer detail can be reached by averaging multiple measurements. When grouping ability estimates into classes, it is more useful to recognize six broader ranges as indicated.

Although the lines in the diagram suggest sharp divisions, the transitions are gradual. They can be compared to mileposts along a road. The landscape does not suddenly change when we pass a milepost; the landscape changes gradually between the mileposts. Yet, mileposts offer useful reference points.

Although the ability scale ends at 0%, this does not mean that even lesser amounts of vision are useless. For the upper ranges of the table, vision (with visual aids, if necessary) is usually sufficient for task performance. For the lower ranges of the table, other senses become increasingly important as an adjunct to vision. For even lower ranges (not shown in the table), residual vision becomes an adjunct to other senses. As an example, consider subjects with bare light perception. They may use their residual vision to determine where the window is in a room. They may then use their memory to

remember where the door is, and their cane skills to reach that door. Their overall performance is based on a combination of skills, each of which would be insufficient in isolation. This makes it hard to assign a % value to each individual skill. Thus, it is reasonable to end the scale for visual skills at 20/2000. Work with visual prosthetics has prompted the development of scales for such rudimentary vision [29], but these are no longer based on the paradigm of letter chart acuity.

Conclusions

While for Snellen, determining visual acuity was an adequate descriptor of vision in general, our concepts of vision have evolved. Today, it is impossible to ignore the distinction between *How each EYE functions*, as measured by letter chart acuity, and *How the PERSON functions,* as measured by task performance.

The use of the logarithmic progression, first proposed by Green (1868) and popularized by the NIH (1982) in combination with the ETDRS chart layout, offers a comprehensive solution that can accommodate all aspects.

References

14 Jaeger E, Schriftskalen, Vienna State Printing House (1854, first edition). Second edition (1857) with texts in German, French, English, Italian, Czech, Russian, Hebrew). Many subsequent editions until 1909.

15 Runge PE. Eduard Jaeger's Test-Types (Schrift-Scalen) and the historical development of vision tests. Trans Am Ophthalmol Soc. 2000; 98: 375–438

16 Donders FC, On the Anomalies of Accommodation and Refraction, New Sydenham Society, London (1864).

17 Donders made his proposal at an 1861 meeting in Heidelberg. Minutes of these meetings, which later evolved into the German Ophthalmological Society, only go back to 1863. Donders mentioned his proposal in his 1862 annual report to the trustees of the Eye Infirmary.

18 de Haan V, Onderzoekingen naar de invloed van de leeftijd op de gezichtsscherpte (Research on the influence of age on visual acuity) – Doctoral Dissertation, Utrecht, (1862)

19 Snellen H, Letterproeven tot Bepaling der Gezichtsscherpte,. Utrecht, Weyers, 1862 (Dutch edition),). Also: Optotypi ad Visum Determinandum. Repeated international editions, incl. 23rd edition, 1937.

20 Green J, On a new series of test-letters for determining the acuteness of vision, Transact. Amer. Opth. Soc., 4th meeting, p 68-71 (1868). Also: Test letters, Green, Ewing. St. Louis, (1886).

20` Preferred Numbers, ISO standard # 3. Also: DIN standard # 323

21 Ferris FL, Kassov A, Bresnick GH, Bailey I, New Visual Acuity Charts for Clinical Research. Am J Ophthalmol 94:91-96 (1982). Also: Measurement Guidelines for Collaborative Studies. National Eye Institute (NEI), Bethesda, MD.

22 Bailey IL, Lovie JE, New Design Principles for Visual Acuity Letter Charts. Am J Optom & Physiol Opt 53:740-745 (1976).

23 Sloan LL, New Test Charts for the Measurement of Visual Acuity at Far and Near Distances. Am J Ophth. 48:807-813 (1959).

24 The mixed contrast reading card, a new screening test for contrast sensitivity. Colenbrander A, Fletcher DC. International Congress Series, 1282:492-497 (Proceedings of Vision-2005 conference, London, 2005).

25 Weber EH. De tactu: annotationices et physiologicae, (1834). Weber described his law for touch. Fechner described broader applicability and brought it to wider attention in: Fechner GT, Elemente der Psychophysik (1860).

26 International Society for Low Vision Research and Rehabilitation (ISLRR), Guide for the Evaluation of Visual Impairment. Pacific Vision Foundation, San Francisco (1999).

27 International Council of Ophthalmology (ICO), Visual Standards, Aspects and Ranges of Vision Loss with emphasis on Population Surveys (2002).

28 American Medical Association, Chicago, Guides to the Evaluation of Permanent Impairment, Chapter 12, The Visual System. AMA press, Chicago. (5th ed. 2001; 6th ed. 2007)

29 HOVER taskforce. Harmonization of Outcomes and Vision Endpoints in Vision Restoration Trials: Recommendations from the International HOVER Taskforce. Trans Vis Sci Tech. 2020;9(8):25,
